# Peripheral whole blood microRNA expression in relation to vascular function: a population-based study

**DOI:** 10.1186/s12967-024-05407-0

**Published:** 2024-07-19

**Authors:** Valentina Talevi, Konstantinos Melas, Gökhan Pehlivan, Mohammed A. Imtiaz, Dennis Manfred Krüger, Tonatiuh Pena Centeno, N. Ahmad Aziz, Andre Fischer, Monique M.B. Breteler

**Affiliations:** 1 Population Health Sciences, German Center for Neurodegenerative Diseases (DZNE), Venusberg-Campus 1, Building 99, 53127 Bonn, Germany; 2 Department for Epigenetics and Systems Medicine in Neurodegenerative Diseases, German Center for Neurodegenerative Diseases, Göttingen, Germany; 3 Bioinformatics Unit, German Center for Neurodegenerative Diseases, Göttingen, Germany; 4 Department of Neurology, Faculty of Medicine, University of Bonn, Bonn, Germany; 5 Department for Psychiatry and Psychotherapy, University Medical Center Göttingen, Göttingen, Germany; 6 Cluster of Excellence “Multiscale Bioimaging: from Molecular Machines to Networks of Excitable Cells” (MBExC), University of Göttingen, Göttingen, Germany; 7 Institute for Medical Biometry, Informatics and Epidemiology (IMBIE), Faculty of Medicine, University of Bonn, Bonn, Germany

**Keywords:** Population-based, Epigenomics, Blood microRNA, microRNA-gene regulatory networks, WGCNA, Vascular function, Arterial compliance, Cardiac output, Biomarkers, White matter hyperintensity

## Abstract

**Background:**

As key regulators of gene expression, microRNAs affect many cardiovascular mechanisms and have been associated with several cardiovascular diseases. In this study, we aimed to investigate the relation of whole blood microRNAs with several quantitative measurements of vascular function, and explore their biological role through an integrative microRNA-gene expression analysis.

**Methods:**

Peripheral whole blood microRNA expression was assessed through RNA-Seq in 2606 participants (45.8% men, mean age: 53.93, age range: 30 to 95 years) from the Rhineland Study, an ongoing population-based cohort study in Bonn, Germany. Weighted gene co-expression network analysis was used to cluster microRNAs with highly correlated expression levels into 14 modules. Through linear regression models, we investigated the association between each module’s expression and quantitative markers of vascular health, including pulse wave velocity, total arterial compliance index, cardiac index, stroke index, systemic vascular resistance index, reactive skin hyperemia and white matter hyperintensity burden. For each module associated with at least one trait, one or more hub-microRNAs driving the association were defined. Hub-microRNAs were further characterized through mapping to putative target genes followed by gene ontology pathway analysis.

**Results:**

Four modules, represented by hub-microRNAs miR-320 family, miR-378 family, miR-3605-3p, miR-6747-3p, miR-6786-3p, and miR-330-5p, were associated with total arterial compliance index. Importantly, the miR-320 family module was also associated with white matter hyperintensity burden, an effect partially mediated through arterial compliance. Furthermore, hub-microRNA miR-192-5p was related to cardiac index. Functional analysis corroborated the relevance of the identified microRNAs for vascular function by revealing, among others, enrichment for pathways involved in blood vessel morphogenesis and development, angiogenesis, telomere organization and maintenance, and insulin secretion.

**Conclusions:**

We identified several microRNAs robustly associated with cardiovascular function, especially arterial compliance and cardiac output. Moreover, our results highlight miR-320 as a regulator of cerebrovascular damage, partly through modulation of vascular function. As many of these microRNAs were involved in biological processes related to vasculature development and aging, our results contribute to the understanding of vascular physiology and provide putative targets for cardiovascular disease prevention.

**Supplementary Information:**

The online version contains supplementary material available at 10.1186/s12967-024-05407-0.

## Background

Perturbation of vascular homeostasis due to vascular dysfunction can lead to cardiovascular diseases (CVD), including peripheral artery disease, coronary syndromes and cerebrovascular diseases [1]. Although several risk factors for CVD are already well-known [2, 3], a more detailed knowledge of the molecular mechanisms underlying vascular function could facilitate the understanding of the pathophysiology of CVD and contribute to the development of more effective preventive and therapeutic interventions.

MicroRNAs are short (18–25 nucleotides) non-coding RNAs, which play a key role in the regulation of gene expression. Their mechanism of action consists of base-pairing with complementary sequences of messenger RNAs (mRNAs), resulting in cleavage of target mRNAs or inhibition of their translation [4]. Due to their ability to bind to multiple mRNAs, a few thousand microRNAs regulate more than 60% of protein-coding genes [5] and are involved in a wide range of physiological and pathological processes [4, 6]. The role of microRNAs in vascular biology and CVD has been extensively studied in recent years. In vitro and animal studies have identified microRNAs implicated in the regulation of endothelial cell homeostasis and angiogenesis, or in the promotion of vascular dysfunction [7, 8]. Examples include miR-145, which is implicated in the regulation of vascular smooth muscle cell proliferation [9, 10], and miR-126 which enhances angiogenesis [11]. In addition, aberrant expression levels of several circulating microRNAs have been observed in CVDs, such as coronary syndromes, heart failure and stroke [12–14]. Circulating microRNAs have garnered increasing attention as promising diagnostic and prognostic biomarkers for CVDs due to their stability in body fluids even under harsh conditions, easy accessibility, and distinctive diseases-related patterns [15]. Taken together, existing evidence points towards an important role of microRNAs in the regulation of vascular function and suggests that microRNA dysregulation contributes to the development and/or progression of CVD.

Despite advances in the field, further characterization of the role of circulating microRNAs in cardiovascular function is needed. The majority of existing research derives from animal models [16] or relatively small observational studies, with a primary focus on specific microRNAs in relation to single vascular traits [17, 18], limiting the understanding of their impact on overall vascular health. Moreover, the limited number of population-based studies employing hypothesis-free approaches has predominantly concentrated on clinical endpoints [12, 13], rather than endophenotypes, which may limit the understanding of disease mechanisms. Additionally, in human studies, the biological functions of microRNAs are usually investigated only *in silico* by querying microRNA target prediction tools, without further functional validation due to the lack of gene expression data [19].

Therefore, in this study we aimed to comprehensively assess the role of microRNAs in vascular function using a three stepped approach. First, we aimed to investigate the circulating blood microRNAs in relation to an extensive panel of quantitative markers of vascular (dys)function (i.e., arterial stiffness, microvascular function and hemodynamics) within the population-based Rhineland Study. This panel included also white matter hyperintensities (WMHs), the most common magnetic resonance imaging (MRI) marker of cerebral small vessel diseases [20]. The prevalence and extent of WMHs strongly increase with age, and they are associated with an increased risk of cognitive decline, dementia and stroke [21]. Including this measurement in the vascular panel allowed for the exploration of potential links among microRNAs, vascular function and brain health. Second, we aimed to analyze microRNA-gene regulatory networks, using gene expression data from the same cohort, to gain insights into the biological functions of microRNAs related to vascular function. Third, we conducted a genome-wide miR-eQTL analysis to corroborate our findings through genetic instruments, specifically by exploring whether specific polymorphisms affect the expression of vascular-related microRNAs.

## Methods

### Data availability

The data from the Rhineland Study are not publicly available due to data protection regulations. Access to data can be provided to scientists in accordance with the Rhineland Study’s Data Use and Access Policy. Requests for additional information or to access to the Rhineland Study’s datasets can be send to RS-DUAC@dzne.de.

### Study population

We used data from the Rhineland Study, an ongoing population-based prospective cohort study in Bonn, Germany. All inhabitants of two geographically-defined areas of Bonn are invited to participate in the Rhineland Study. There are no specific selection criteria. The only requirements are that participants are aged 30 years and above, and have sufficient command of the German language to provide informed consent.

Approval to undertake the study was obtained from the ethics committee of the University of Bonn, Medical Faculty. The study is carried out in accordance with the recommendations of the International Conference on Harmonization Good Clinical Practice standards. We obtained written informed consent from all participants in accordance with the Declaration of Helsinki.

In this study, we used baseline data of the first 3000 consecutive participants of the Rhineland Study. MicroRNA expression data were unavailable for 33 individuals due to technical issues, with an additional 38 participants being excluded during quality control procedures. Moreover, 323 participants were excluded because of missing cardiovascular data due to contraindication/exclusion criteria (*n* = 246), participant refusal (*n* = 8), technical problems (*n* = 12), exclusion during quality assurance (*n* = 51) and for other/unknown reasons (*n* = 6). Our final analysis was conducted in a subset of 2606 participants for whom both microRNA expression data and cardiovascular measurements were available.

### Vascular function measurements

#### Blood pressure

Systolic blood pressure (SBP) and diastolic blood pressure (DBP) were measured in a sitting position, using an oscillometric blood pressure device (Omron 705 IT) [22, 23]. The measurements were performed thrice, separated by ten minutes, by experienced study technicians while participants were sitting in a resting chair in a quiet environment. Cuff size was determined by measuring participants’ arm circumference in the middle of the upper arm between the acromion and olecranon on the right arm of the participant sitting in the measuring position. Measurements were preferably performed in the right arm. In cases where the measurements were not possible on the right arm, the left arm was used. The measured arm was always placed in a resting position at heart level, with the palms facing upward, the shoulders in a horizontal position, and both legs resting on the ground. The mean of the second and third measurement was used in the analyses. Mean arterial pressure (MAP) was calculated as (SBP + 2 × DBP)/3 [24].

#### Hemodynamic measurements

Impedance cardiography was performed with the CardioScreen 2000 device (Medis, Germany), by experienced study technicians, in a temperature-controlled room. Before the examination, the participants were placed in a supine position and allowed to rest for five minutes. Electrodes as well as arm, ankle and thigh cuffs were placed as per the device manufacturer’s recommendations [25]. All hemodynamic measurements were calculated by in-developed software, based on simultaneously registered electrocardiography signals and blood pressures with 2-minute intervals. We set central venous pressure (CVP) at 6 mmHg as recommended by the producer. Whereas normal CVP reportedly can vary from 5 to 15 mmHg [26], a recent study has shown that setting CVP at certain values does not impact systemic vascular resistance-related outcomes [26].

Stroke volume (mL) was defined as the volume of blood pumped from the left ventricle to systemic circulation in each heartbeat and computed using beat-to-beat for approximately 8 min with an impedance cardiography device (CardioScreen 2000, Medis, Germany) [27]. Cardiac output (L/min) was calculated as stroke volume multiplied by heart rate (beats per minute), and systemic vascular resistance (dynes/sec/cm^5^) as MAP divided by cardiac output, multiplied by 80. We divided stroke volume, cardiac output and systemic vascular resistance by body surface area (BSA, m^2^), calculated based on the participant’s height and weight using the Du Bois formula [28], to obtain the stroke index (mL/m^2^), cardiac index (L/min/m^2^) and systemic vascular resistance index (dynes/sec/cm^5^/m^2^), respectively.

#### Arterial stiffness measurements

As measures of arterial stiffness, we included aorta-femoral pulse wave velocity (m/s) and total arterial compliance (mL/mmHg). The propagation time of the pulse wave was defined as the difference between the opening of the aortic valve, assessed through impedance cardiography waves, and the arrival of the pulse wave to the mid-femoral cuff. We calculated the pulse wave velocity as the ratio between the distance between the supra-sternal notch and the mid-femoral cuff and the propagation time. Total arterial compliance was obtained by dividing stroke volume by pulse pressure, which is the difference between SBP and DBP. To obtain the total arterial compliance index (mL/mmHg/m^2^), total arterial compliance was divided by BSA.

#### Microvascular function

Microvascular function was measured through the reactive skin hyperemia parameter. The examination lasted a total of 26 min, during which time the skin blood flow was measured on the ventral surface of the forearm through a laser Doppler flowmetry device (Moors, UK). In the first 2 min, the skin blood flow at baseline was measured. Afterwards, the temperature in the defined region was increased to 40 °C using a heating probe (following a local thermal heating protocol). Reactive skin hyperemia was defined as the percentage increase between the skin blood flow at baseline and the last 2 min of the plateau level.

#### White matter hyperintensities

WMHs in the brain were assessed through 3T MRI, which included T1- and T2-weighted, and fluid-attenuated inversion recovery (FLAIR) sequences. WMHs were determined as the hyperintense signal of the white matter tracts in the supratentorial region on T2-weighted images, automatically outlined using an in-house developed algorithm based on DeepMedic [29], utilizing image information from T1- and T2-weighted, and FLAIR sequences. A subset of the automatically segmented images was manually checked for quality assurance. White matter volume was extracted using the FreeSurfer automated segmentation pipeline [30]. To account for the effect of brain size, WMH burden was defined as the ratio of WMHs to white matter volume.

### MicroRNA and gene expression profiling

Fasting blood samples were collected between 7:00 to 9:45 in the morning from an antecubital or dorsal hand vein and stored in PAXgene Blood RNA tubes (PreAnalytix/Qiagen). Total RNA was isolated according to the manufacturer’s instructions using PAXgene Blood miRNA Kit and following the semi-automatic purification protocol (PreAnalytix/Qiagen). NEBNext® small RNA library preparation kit was used to generate sequencing libraries. Briefly, we used 100 ng of isolated total RNA as starting material for adapter ligation, primer hybridization, cDNA generation. 150 base pairs band was cut and chosen as insert size for library quantification. The microRNA sequencing was performed on the Illumina HiSeq 2000 platform using a 50-bp single read setup. The quality of the sequencing reads was evaluated through FastQC v0.11.9 software. Sequencing adapters, low-quality score reads (i.e., reads with the average quality per 4-base wide below 15) and reads shorter than 18 base pairs were discarded using Trimmomatic v0.39 software. The alignment and the quantification of microRNA data were performed by Mirdeep2 v2.0.1.2 tool [31], using Human Genome GRCh38.p13 provided by Ensembl and microRNA mature and precursor sequences obtained from miRBase v22.1 [32].

The gene expression profiling was performed on 750 ng of isolated total RNA. After checking RNA integrity and quantity (TapeStation 4200, Agilent), NGS libraries for total RNA sequencing were generated (TruSeq stranded total RNA kit with Ribo-Zero Globin, Illumina). Library size distribution (D1000 assay on TapeStation 4200, Agilent) and quantification (Qubit HS dsDNA assay, Invitrogen) were checked and libraries were clustered at a final 250 pM concentration. The sequencing was performed in paired-end mode (2*50 cycles) on a NovaSeq6000 instrument (Illumina) using S2 v1 chemistry in XP mode, followed by demultiplexing using bcl2fastq2 v2.20. After checking the reads sequence quality with FastQC v0.11.9, the sequencing reads were aligned to the human reference genome GRCh38.p13 provided by Ensembl using STAR v2.7.1 [33]. The count matrix was generated with STAR –quantMode GeneCounts using the human gene annotation version GRCh38.101.

MicroRNAs and genes with an overall mean expression greater than 15 reads and expressed in at least 5% of the participants were used for subsequent analyses. Finally, to normalize for library size and to log-transform the raw data, we applied the varianceStabilizingTransformation function from DESeq2 v1.30.1 R package [32].

### Genetics profiling

Genotyping was performed on the Omni 2.5 Exome Array, while GenomeStudio (version 2.0.5) was used for genotype calling. Quality control was performed through PLINK (version 1.9) by checking for poor genotyping rate (< 99%), Hardy-Weinberg disequilibrium (*p* < 1e − 6), poor sample call rate (< 95%), abnormal heterozygosity, cryptic relatedness, and sex mismatch. Population sub-structure was analyzed through EIGENSOFT v7.2.1.0 [34]. To account for systematic variations in allele frequencies due to different ethnic backgrounds, we excluded participants of non-Caucasian descent. Genotype imputation was performed using IMPUTE v2 [35] with 1000 Genomes phase 3 v5 as the reference panel [36].

### Demographic and health variables

Information on age and sex was collected through a questionnaire. Body mass index (BMI) was calculated as weight (kg) divided by height squared (m^2^). Cardiovascular conditions, including stroke, heart failure, coronary artery disease, arrhythmia, heart valve disease, myocardial infarction and peripheral arterial disease were defined as a self-reported physician diagnosis. Hypertension status was defined based on the mean measured systolic blood pressure ≥ 140 mmHg and/or diastolic blood pressure ≥ 90 mmHg, or antihypertensive drug use. Differential blood cell counts (i.e., erythrocytes, leukocytes, and platelets) were assessed at the Central Laboratory of the University Hospital in Bonn, using EDTA-whole blood samples on a hematological analyzer Sysmex XN9000.

### Statistical analysis

#### Weighted microRNA co-expression network analysis

We clustered microRNAs with highly correlated expression levels with the weighted gene co-expression network analysis (WGCNA) method, using the WGCNA R package v1.70-3 [37]. The WGCNA approach assumes that microRNAs that present similar expression patterns tend to be involved in similar biological functions [38]. The clustering analysis was conducted on residual microRNA levels after regressing out the effects of sequencing batch. Firstly, the adjacency matrix was constructed using the threshold power of 7, which was the first power value to reach 0.90 in the scale-free topology and corresponded to a relatively low mean connectivity value [37] (Additional file 1: Fig. S1). The similarity matrix was generated based on biweight midcorrelation and with the signed parameter. The topological overlap matrix (TOM) and the corresponding dissimilarity (1-TOM) were then calculated. MicroRNA modules were identified by first generating a hierarchical clustering tree using the “average” algorithm and then by clustering microRNAs using the Hybrid Dynamic Tree Cut method, setting the minimum cluster size to 5. Finally, highly correlated modules were merged using 0.2 as the “cutHeight” parameter, corresponding to a correlation coefficient of 0.8. We then used the module eigen-microRNA vector, calculated as the first principal component of each module, to represent the expression profile of all microRNAs within a given module. Moreover, we computed the module membership measurement for each microRNA, which describes the correlation between a given microRNA’s expression level and the corresponding module eigen-microRNA vector [37].

#### Identification of key modules related to vascular function

Before further analysis, we applied a log-transformation for pulse wave velocity and reactive skin hyperemia and a logit transformation for WMH burden to account for their skewed distributions. All the numerical variables were standardized to a mean of 0 and a standard deviation of 1 to allow for better comparison of the effect sizes across the different cardiovascular traits.

As a first exploratory analysis, we evaluated the relationship between age and cardiovascular measurements, as well as microRNA modules.

Next, to identify key modules related to vascular function, we investigated the association of module eigen-microRNA vectors (independent variables) with indexed cardiovascular measurements and WMH burden (dependent variables), by running multivariable linear regression models, adjusting for age and sex.

To examine whether indexing the cardiovascular measurements for BSA affected our results, we also conducted a sensitivity analysis using the non-indexed cardiac output, stroke volume, total arterial compliance and systemic vascular resistance measurements as dependent variables, adjusting for age and sex. Moreover, we examined whether our results were affected when adjusting for BMI rather than BSA, by additionally including BMI as a covariate in the analysis of the non-indexed measurements.

Multiple testing correction was applied using the Benjamini-Hochberg false discovery rate (FDR) method [39]. We reported results both at the FDR < 0.1 and FDR < 0.05 thresholds. This was done to avoid rejecting as statistically non-significant results that were very close to the common FDR < 0.05 threshold, thus reducing the possibility of false negatives (Type II error).

#### Identification of hub-microRNAs for each module

For each key module, defined as a module significantly associated with at least one of the investigated traits, hub-microRNAs were identified. We defined hub-microRNAs as microRNAs that *(a)* had module membership greater than 0.8, and *(b)* were significantly associated with the selected trait (*p*-value < 0.05). Therefore, hub-microRNAs served as module representatives and were the main drivers of the association between the module and the candidate trait.

#### Mediation analysis

Additionally, we investigated whether the associations between module eigen-microRNA vectors or hub-microRNA expression levels (exposures) and WMH burden (outcome) were mediated through cardiovascular function. We performed structural equation modeling using the R package laavan v0.6-11. The mediation models were adjusted for age and sex, with the threshold for statistical significance set at *p*-value < 0.05.

#### Age and sex modifications

For each hub-microRNA significantly associated with a trait, we assessed whether the association was modified by age or sex through interaction analyses. Specifically, we used linear regression models, adjusting for age and sex and including interaction terms of hub-microRNA with age and, in separate models, with sex. In case of significant interaction terms (*p*-value < 0.05), we conducted further stratified analyses by age quartiles and sex.

#### Exploratory analysis of hub-microRNAs

To investigate the overall role of hub-microRNAs in cardiovascular health, they were also evaluated in relation to all other cardiovascular traits. The regression models were adjusted for age and sex and the statistical significance threshold was set at *p*-value < 0.05.

#### Hub-microRNA – target genes and functional analysis

To map genes that are potentially regulated by the identified hub-microRNAs, we integrated microRNA and gene expression data. The analysis was conducted on 2181 participants of the Rhineland Study for which microRNA expression data, gene expression data and blood cell counts were available. First, we collected data on experimentally validated target genes and predicted target genes using the R package multiMir v1.12.0. Specifically, we queried MirTarBase [40] and TargetScan [41], widely recognized online databases for microRNA target gene prediction, and miRDB [42] a database providing experimentally validated microRNA-target interactions. Subsequently, we confirmed the relation between each putative microRNA-target gene pair. For this, after correction of microRNA and gene expression levels for batch effects, we created a model using gene expression levels as the dependent variable and microRNA expression levels as the main independent variable, adjusting for age, sex, erythrocyte, leukocyte, and platelet counts. A confirmed target gene was defined as negatively associated (beta estimate < 0 and *p*-value < 0.05) with the targeting microRNA.

To gain insights into the biological processes regulated by the hub-microRNAs and the corresponding target genes, we performed over-representation analyses on confirmed target genes, separately for each key module. Specifically, we first determined overrepresented Gene Ontology: Biological Process (GO: BP) terms, using the R package clusterProfiler v3.18.1. Next, we aggregated semantically similar terms to facilitate the identification of key terms through rrvgo v1.2.0. Briefly, rrvgo computes the semantic similarity matrix between pairs of GO terms and aggregates them together through complete linkage. We set a low similarity threshold of 0.5 to only merge highly similar terms. The *p*-values of the terms within each group were combined using the Fisher method and multiple testing correction was applied using the Benjamini-Hochberg false discovery rate (FDR) method [39]. Only terms that reached the significant level at FDR < 0.05 were reported.

#### Genome-wide miR-eQTL analysis

To identify potential microRNA expression quantitative trait loci (miR-eQTLs), we conducted a genome-wide miR-eQTL analysis on 2456 participants of the Rhineland Study with both genetic and microRNA expression data available. Specifically, we first adjusted microRNA expression levels for age, sex, the first 10 genetic principal components and batch effects, and extracted the residuals. Subsequently, linear regression analysis was used to assess the relation between each SNP (independent variable) and these hub-microRNA residuals (dependent variable). *Cis*-SNPs were defined as those located within 1 Mb of the mature microRNA, whereas trans-SNPs were defined as those positioned elsewhere [43]. The genome-wide significance level for miR-eQTLs was set at *p*-value < 5e-8. SNPs with relatively high linkage disequilibrium (i.e., r2 > 0.6) with nearby SNPs were clumped to define genomic risk loci, using the Functional Mapping and Annotation of Genome-Wide Association Studies (GWAS) platform [44]. Lead SNPs in these genomic risk loci were defined as those independent significant SNPs that were in approximate linkage disequilibrium with each other at r2 < 0.1.

## Results

### Participant characteristics

The characteristics of the study population are reported in Table 1. The analysis included 2606 participants with available microRNA expression data and cardiovascular measurements. The mean (± standard deviation) age of the participants was 53.93 ± 13.95, including 1194 men (45.8%). Among the participants included in the study, the following cardiovascular conditions were self-reported: stroke (*n* = 50), heart failure (*n* = 65), coronary artery disease (*n* = 145), hypertension (*n* = 1076), arrhythmia (*n* = 336), heart valve disease (*n* = 107), myocardial infarction (*n* = 54) and peripheral arterial disease (*n* = 40). A smaller dataset (*N* = 2024) that largely but not entirely overlapped (Additional file 1: Fig. S2) was queried to investigate the relation of microRNA expression levels with WMH burden, including all participants for which microRNA and WMH data were available. There were no significant differences in population characteristics between these two datasets (Table 1). Moreover, the subsample (*N* = 2182) used to conduct the integrative analysis of microRNA and gene expression data was similar to the initial dataset, with an average age of 54.85 ± 14.39 and including 987 men (45.2%) (Additional file 1: Fig. S2).

**Table 1:** Characteristics of the study population

Characteristics	Participants with complete cardiovascular traits and microRNA data *N* = 2606^a^	Participants with complete white matter hyperintensities and microRNA data *N* = 2024	*p*-value^b^
Age (years)	53.93 ± 13.95	53.95 ± 14.02	0.956
Sex = men (%)	1194 (45.8)	878 (43.4)	0.104
Pulse wave velocity (m/s)	6.86 ± 3.27	6.78 ± 2.18	0.348
Total arterial compliance index (mL/mmHg/m^2^)	1.04 ± 0.25	1.05 ± 0.25	0.350
Stroke index (mL/m^2^)	51.83 ± 8.51	51.90 ± 8.59	0.776
Cardiac index (L/min/m^2^)	3.19 ± 0.52	3.20 ± 0.52	0.786
Systemic vascular resistance index (dynes/sec/cm^5^/m^2^)	2121.85 ± 467.29	2114.73 ± 450.87	0.611
Reactive skin hyperemia (%)	5.87 ± 0.94	5.88 ± 0.93	0.881
Diastolic blood pressure (mmHg)	76.2 ± 9.3	76.2 ± 9.3	0.896
Systolic blood pressure (mmHg)	127.0 ± 16.0	127.0 ± 15.9	0.979
Mean arterial pressure (mmHg)	93.1 ± 10.6	93.1 ± 10.6	0.950
White matter hyperintensity burden supratentorial	/	0.0038 ± 0.0096	
Erythrocyte count (T/L)	4.69 ± 0.43	4.68 ± 0.44	0.388
Leukocyte count (G/L)	5.68 ± 1.54	5.67 ± 1.54	0.835
Platelet count (G/L)	238.43 ± 53.83	238.91 ± 54.70	0.774

The data are expressed as mean ± standard deviation for continuous variables and as number (percentage) for categorical variables

^a^ Participants with missing data: pulse wave velocity: *n* = 23; total arterial compliance index, systemic vascular resistance index: *n* = 4

^b^ The differences between the two datasets were calculated using a t-test (continuous) or chi-squared test (categorical)

### Weighted microRNA co-expression network analysis

**Fig. 1:**
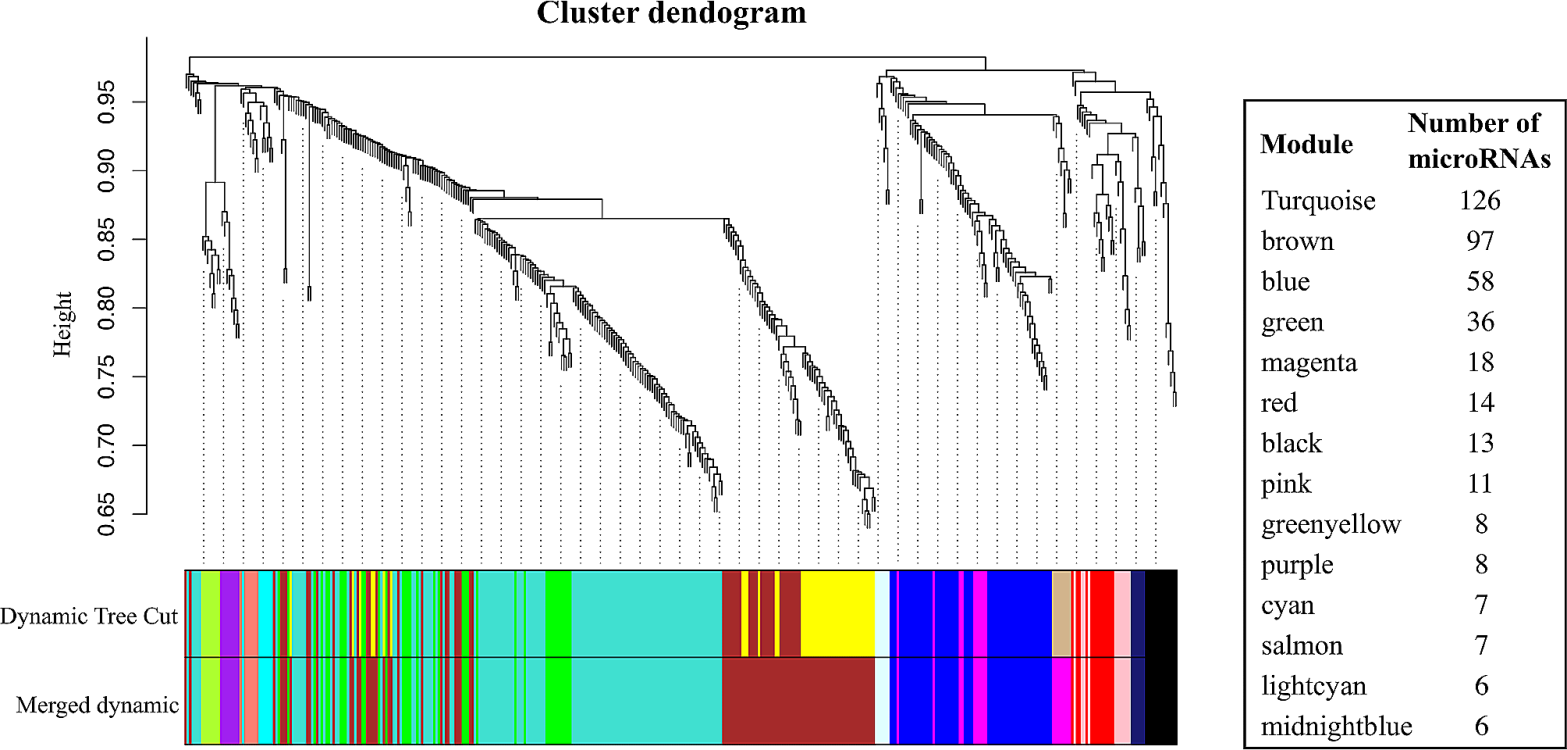
Identification of distinct modules of co-expressed microRNAs using weighted gene co-expression network analysis (WGCNA) Dendrogram showing the clustering of microRNAs before and after the merging step, together with the module colors and the number of microRNAs belonging to each module

We applied a clustering approach to identify modules (i.e., clusters) of co-expressed microRNAs associated with cardiovascular traits. Specifically, WGCNA method clustered 415 microRNAs, for which the expression levels were detectable within the Rhineland Study cohort, into 14 modules. Random color-names were then assigned to each module. The largest module (*turquoise*) included 126 microRNAs, while the smallest ones (*lightcyan* and *midnightblue*) had 6 microRNAs (Fig. 1, Additional file 2: Table S1).

### Identification of key modules related to vascular function

The exploratory analysis showed the expected relationships between age and vascular measurements: total arterial compliance index, cardiac index, stroke index and reactive skin hyperemia decreased with age, while pulse wave velocity, systemic vascular resistance index and WMH burden increased. In contrast, the eigen-microRNA vectors were relatively stable across age (Additional file 1: Fig. S3).

**Fig. 2:**
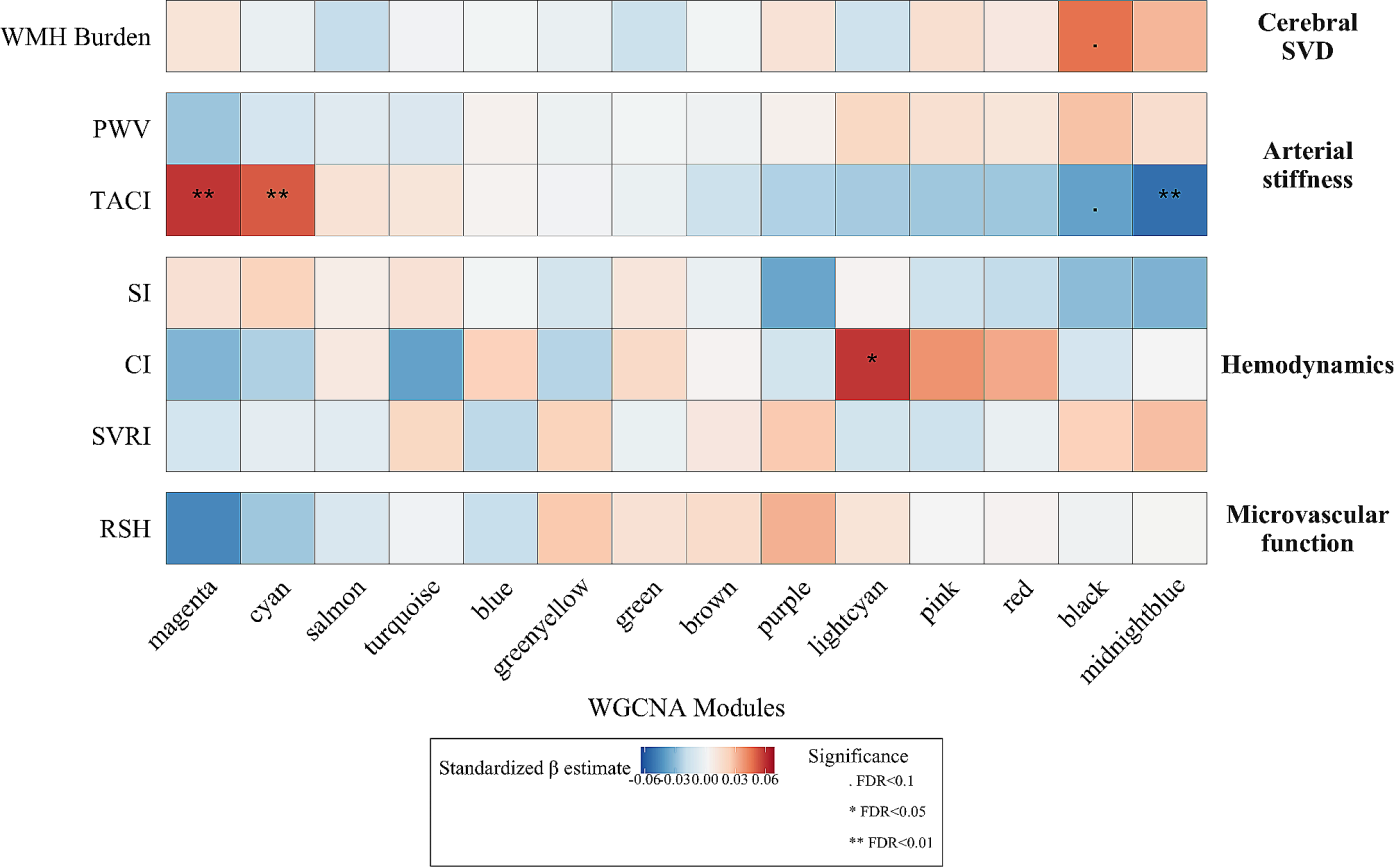
Relation between module eigen-microRNA vectors and quantitative markers of vascular function Heatmap showing the relations between module eigen-microRNA vectors (columns) and indexed cardiovascular traits and white matter hyperintensity burden (rows) assessed by multivariable linear models, adjusting for age and sex. Abbreviations: CI, cardiac index; PWV, pulse wave velocity; RSH, reactive skin hyperemia; SI, stroke index; SVD, small vessel disease; SVRI, systemic vascular resistance index; TACI, total arterial compliance index; WMH, white matter hyperintensities

Next, we investigated the association between microRNA modules and cardiovascular measurements. We observed that higher eigen-microRNA vectors of the *magenta* and *cyan* modules were associated with better total arterial compliance index (β estimate = 0.059, FDR = 0.004 and β estimate = 0.052, FDR = 0.007, respectively), while higher eigen-microRNA vectors of the *black* and *midnightblue* modules were associated with a worse total arterial compliance index (β estimate = -0.039, FDR = 0.063 and β estimate = -0.054, FDR = 0.007, respectively). Consistent with the above results, we found a larger eigen-microRNA vector of the *black* module to be associated with an increase in WHM burden (β estimate = 0.047, FDR = 0.053). Furthermore, we observed a positive association between eigen-microRNA vector of the *lightcyan* module and cardiac index (β estimate = 0.059, FDR = 0.020) (Fig. 2, Additional file 2: Table S2).

Sensitivity analysis, conducted on non-indexed cardiovascular measurements and additionally adjusting for BMI, showed similar but weaker associations, confirming the importance of the abovementioned key modules. The only exception was the cardiac output trait, as the eigen-microRNA vectors of the *magenta*, *cyan*, *lightcyan* and *pink* modules were significantly associated with the non-indexed value, but not with the indexed value. However, all the associations became non-significant after adjustment for BMI, indicating confounding by body size (Additional file 1: Fig. S4, Additional file 2: Table S3).

### Identification of hub-microRNAs for each module

**Fig. 3:**
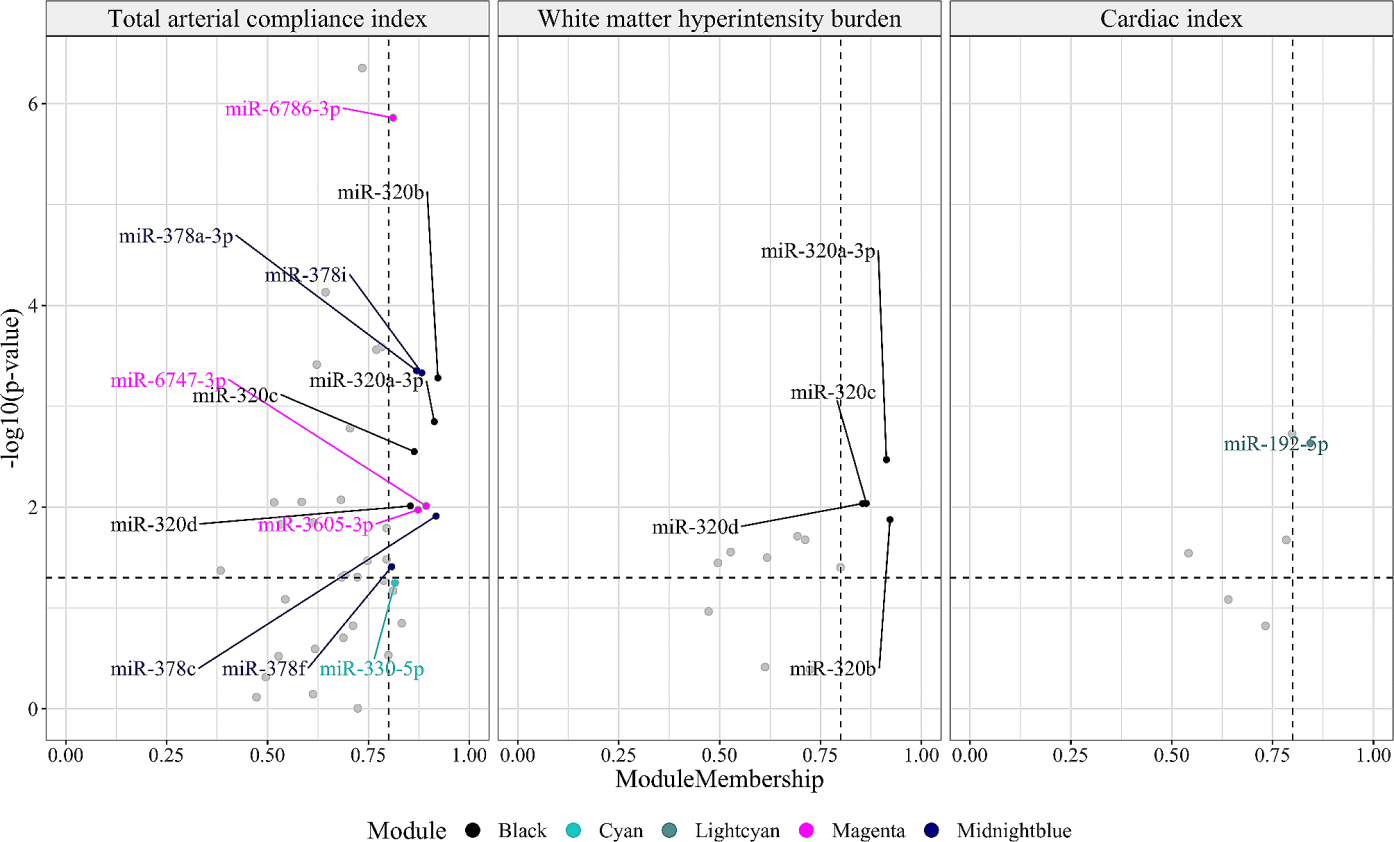
Hub-microRNAs of key modules related to vascular function Identification of hub-microRNAs in *black*, *cyan*, *lightcyan*, *magenta* and *midnightblue* modules. The hub-microRNAs are defined as microRNAs that have module membership greater than 80 and are significantly associated with the selected trait (*p*-value < 0.05)

Next, we detected the hub-microRNAs for each key module, defined as any microRNAs that had high module membership values and were significantly associated with the corresponding trait, as depicted in Fig. 3 (Table 2).

**Table 2:** Relation between hub-microRNAs and total arterial compliance index, white matter hyperintensity burden and cardiac index

Trait	microRNA	β^a^ [95% CI]	*p*-value	Modules	Module membership
Total arterial compliance index	miR-320a-3p	-0.052 [-0.083; -0.020]	0.001	black	0.913
	miR-320b	-0.056 [-0.088; -0.025]	5.24e-04	black	0.922
	miR-320c	-0.048 [-0.080; -0.017]	0.003	black	0.864
	miR-320d	-0.042 [-0.074; -0.010]	0.01	black	0.854
	miR-378a-3p	-0.058 [-0.090; -0.026]	4.432e-04	midnight-blue	0.869
	miR-378c	-0.041[-0.073; -0.009]	0.012	midnight-blue	0.918
	miR-378f	-0.034 [-0.066; -0.002]	0.039	midnight-blue	0.808
	miR-378i	-0.058 [-0.090; -0.025]	4.657e-04	midnight-blue	0.882
	miR-3605-3p	0.041 [0.010; 0.073]	0.011	magenta	0.873
	miR-6747-3p	0.042 [0.010; 0.074]	0.01	magenta	0.893
	miR-6786-3p	0.078 [0.046; 0.109]	1.378e-06	magenta	0.811
	miR-330-5p	0.031 [-0.001; 0.063]	0.056	cyan	0.816
White matter hyperintensity burden	miR-320a-3p	0.048 [0.016; 0.080]	0.003	black	0.913
	miR-320b	0.040 [0.008; 0.072]	0.013	black	0.922
	miR-320c	0.042 [0.010; 0.073]	0.009	black	0.864
	miR-320d	0.043 [0.011; 0.07]	0.009	black	0.854
Cardiac index	miR-192-5p	0.056 [0.020; 0.092]	0.002	lightcyan	0.843

^a^ Change in standard deviations in vascular outcome measures (total arterial compliance index/white matter hyperintensity burden/cardiac index) per one standard deviation change in microRNA expression level

Abbreviation: 95% CI, 95% confidence interval

Members of the miR-320 family (miR-320a-3p, miR-320b, miR-320c and miR-320d) were identified as hub-microRNAs for the *black* module, as they had a high module membership and were associated with both total arterial compliance index and WMH burden.

Similarly, for the modules related only to total arterial compliance index, we identified four hub-microRNAs belonging to the miR-378 family (miR-378a-3p, miR-378c, miR378f and miR-378i) for the *midnightblue* cluster and three hub-microRNAs (miR-6786-3p, miR-6747-3p and miR-3605-3p) for the *magenta* cluster.

Within the *cyan* module, none of the microRNAs fulfilled the criteria to be defined as a hub-microRNA. However, to be able to further explore this module, which was significantly associated with total arterial compliance index, we considered miR-330-5p as the hub-microRNA. This selection was based on its proximity to satisfying both hub-microRNA criteria (i.e., module membership and association with the outcome). Specifically, miR-330-5p had the highest module membership value (0.816) and it showed a borderline significant association with total arterial compliance index (*p*-value = 0.056).

Finally, miR-192-5p, which was significantly associated with cardiac index, was defined as the hub-microRNA for the *lightcyan* module.

### Mediation analysis

**Fig. 4:**
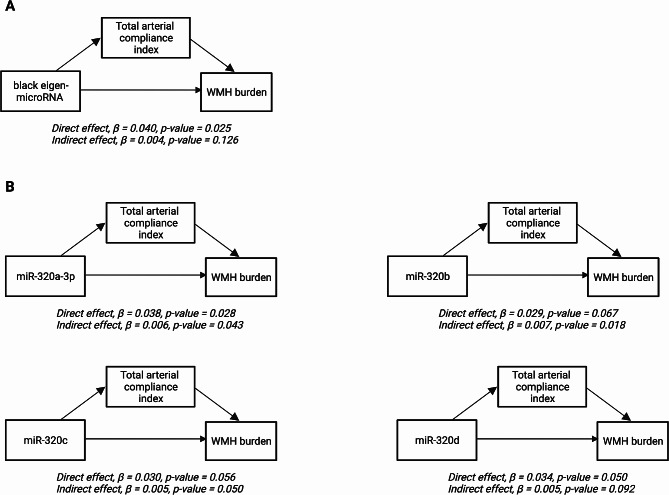
Mediation analysis models (**A**) Mediation analysis model for the relationship between black eigen-microRNA vectors and white matter hyperintensity burden, mediated by total arterial compliance index. (**B**) Mediation analysis models for the relationships between individual *black* hub-microRNAs and white matter hyperintensity burden, mediated by total arterial compliance index. All the models are adjusted for age and sex. Abbreviation: WMH, white matter hyperintensity

The previous analyses showed an association between the *black* module and both the total arterial compliance index and WMH burden. Based on that, we hypothesized that the effect of *black* eigen-microRNA vector or *black* hub-microRNAs on WMH burden was mediated by total arterial compliance index. We examined this using mediation analysis. We treated *black* eigen-microRNA vector or *black* hub-microRNAs as the exposure, WMH burden as the outcome and total arterial compliance index as the mediator (Fig. 4). We found that 18.8%, 14.3% and 13.1% of the effects of miR-320b, miR-320c and miR-320a-3p, respectively, on WMH burden were mediated by the total arterial compliance index (Additional file 2: Table S4).

### Age and sex modifications

Next, we assessed whether the associations of the 13 hub-microRNAs with cardiovascular traits were modified by age or sex. We found that age modifies the association of miR-378f, miR-378i, miR-3605-3p, miR-6747-3p, miR-6786-3p and miR-330-5p with total arterial compliance index (Additional file 2: Table S5). Specifically, we observed that the effects of miR-3605-3p, miR-6747-3p and miR-6786-3p were stronger in younger (30–43 and 44–53 years) compared to older (54–65 and 66–95 years) participants. In contrast, miR-378f, miR-378i and miR-330-5p had larger effect sizes on total arterial compliance index in the middle-aged group (44–53 years) compared to younger (30–43 years) and older (54–65 and 66–95 years) participants (Additional file 1: Fig. S5-A, Additional file 2: Table S6). Furthermore, we found that the association between miR-320d and total arterial compliance index was modified by sex (Additional file 1: Fig. S5-B, Additional file 2: Table S7). After sex stratification, lower miR-320d expression levels were associated with better total arterial compliance in women, whereas in men the association was negligible (Additional file 2: Table S8).

### Exploratory analysis of hub-microRNAs

Furthermore, we evaluated the role of the 13 hub-microRNAs in cardiovascular health, investigating their relation to all other cardiovascular traits. We observed that higher expression levels of miR-320 family were also associated with higher values of pulse wave velocity, systemic vascular resistance index and SBP. Similarly, higher expression values of miR-378 family were associated with higher blood pressure. Interestingly, miR-6786-3p expression levels were associated with most of the markers of vascular function (i.e., pulse wave velocity, cardiac index, reactive skin hyperemia and all the blood pressure measurements) (Additional file 1: Fig. S6, Additional file 2: Table S9).

### Hub-microRNA – target genes and functional analysis

To gain insights about the biological role of the microRNAs, we leveraged gene expression data. An overview of the number of predicted and confirmed target genes of the identified hub-microRNAs is reported in Table 3 and Additional file 2: Table S10. Specifically, between 19% and 34.4% of the target genes predicted on the basis of three online databases (MirTarBase, TargetScan and miRDB) were confirmed in our integrative microRNA-gene expression analysis. Interestingly, we found 76 genes commonly targeted by miR-6747-3p (*magenta*) and miR-330-5p (*cyan*), and 33 genes commonly targeted by miR-6747-3p (*magenta*) and miR-192-5p (*ligthcyan*) (Additional file 1: Fig. S7, Additional file 2: Table S10

**Table 3:** Number of target genes for each hub-microRNA

Hub-microRNAs	Predicted target genes^a^	Confirmed target genes^b^	Percentage of confirmed genes
miR-320b	1621	502	31.0
miR-320c	1572	481	30.6
miR-320d	1540	477	31.0
miR-330-3p	1330	458	34.4
miR-6747-3p	1351	414	30.6
miR-320a-3p	1069	358	33.4
miR-192-5p	1311	260	19.8
miR-378a-3p	619	155	25.0
miR-378c	494	118	23.9
miR-378i	488	115	23.6
miR-378f	489	110	22.5
miR-6786-3p	52	13	25.0
miR-3605-3p	58	11	19.0

^a^ Obtained from three online databases (MirTarBase, TargetScan and miRDB).

^b^ Predicted target genes whose associations were replicated in the Rhineland Study cohort. They are defined as genes negatively associated (*p*-value < 0.05) with the corresponding targeting microRNAs in separate linear regression models adjusted for age, sex and blood cell counts

**Fig. 5:**
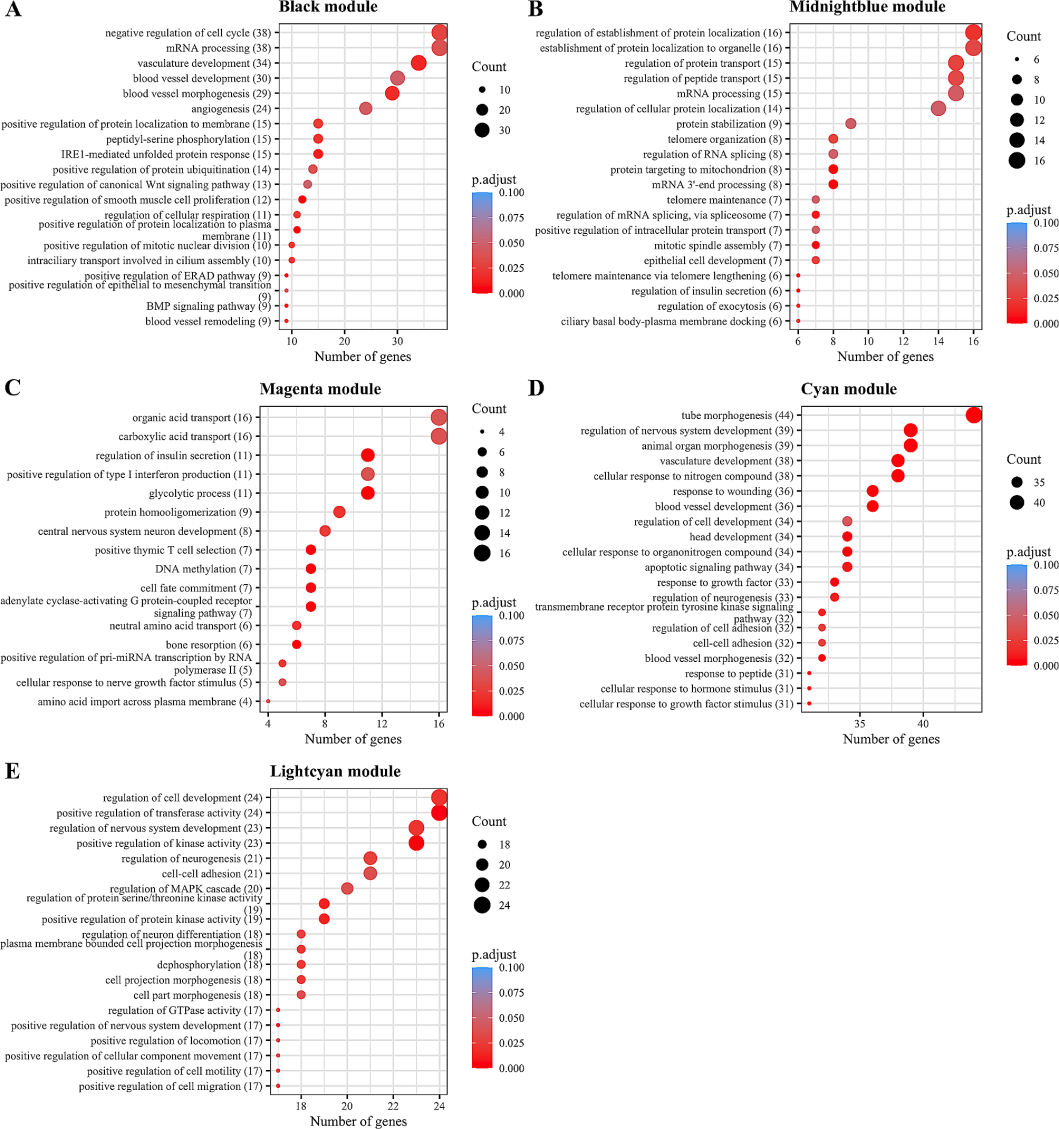
Functional enrichment analysis on confirmed target genes Functional enrichment analysis was run on the validated target genes, separately for each module. (**A**) Top-20 Gene Ontology: Biological Process (GO: BP) for confirmed target genes (*N* = 529) of hub-microRNAs in the *black* module. (**B**) Top-20 GO: BP for confirmed target genes (*N* = 159) of hub-microRNAs in the *midnightblue* module. (**C**) GO: BP pathways for confirmed target genes (*N* = 434) of the hub-microRNA in the *magenta* module. (**D**) Top-20 GO: BP pathways for confirmed target genes (*N* = 458) of the hub-microRNA in the *cyan* module. (**E**) Top-20 GO: BP pathways for confirmed target genes (*N* = 260) of the hub-microRNA in the *lightcyan* module

Functional analysis, run separately for each module, revealed several important pathways related to cardiovascular function (Fig. 5, Additional file 2: Table S11). In particular, for two modules related to total arterial compliance index (i.e., *black* and *cyan*), we found enriched target genes in pathways related to blood vessel development and morphogenesis, as well as angiogenesis, including genes previously reported to be related to arterial stiffness (*TGFBR1, TGFB1* [45], *HIF1A* [46], and *AKT1* [47]). Additionally, among the pathways overrepresented in the other two modules associated with total arterial compliance index, we identified telomere organization, epithelial cell development processes (*midnightblue*), type I interferon production and insulin secretion pathways (*magenta*). Lastly, cell-cell adhesion and regulation of mitogen-activated protein kinase (MAPK) cascade biological processes were enriched in the *lightcyan* module, which was associated with cardiac output.

### Genome-wide miR-eQTL analysis

Finally, we conducted a genome-wide miR-eQTL analysis for the 13 hub-microRNAs to determine whether certain genetic variants might impact microRNA expression. We identified 136 GWAS *cis*-signals for miR-3605-3p on chromosome 1, with the top lead SNP (rs11554674, β estimate = -0.173, GWAS *p*-value = 6.035e-14) mapping to the *PHC2* gene. This finding indicates a strong genetic influence on the expression levels of miR-3605-3p. Moreover, GTEx v8 data revealed that rs11554674 is an expression quantitative trait loci for *A3GALT2*, an enzyme involved in sphingolipid metabolism, in several tissues, including whole blood (*p* = 1.0e-44, NES = 0.49) and artery – aorta tissue (*p* = 6.2e-33, NES = 0.89) [48]. Finally, 4 *trans*-miR-eQTLs were detected for miR-320a-3p, miR-miR-320c and miR-6747-3p (Additional file 2: Table S12).

## Discussion

We investigated the relation of peripheral whole blood microRNA expression levels and quantitative markers of vascular health in a large population-based cohort spanning a wide age range. We identified associations with total arterial compliance for four microRNA modules, represented by the miR-320 family, miR-378 family, miR-6786-3p, miR-6747-3p, miR-3605-3p and miR-330-5p. Importantly, our results showed that the miR-320 family module was also associated with WMH burden. Finally, we found one module, represented by miR-192-5p, to be related to cardiac index.

Our finding that higher expression levels of miR-320 family members were associated with worse total arterial compliance is in line with previous studies. The miR-320 family has been widely investigated in the context of cardiovascular health and is involved in several biological processes related to vascular (dys)function. Using in vitro and in vivo experimental models, it has been shown that overexpression of mir-320a and miR-320b enhances atherosclerosis through endothelial and vascular smooth muscle cell dysfunction, and plaque formation [49–51]. Aberrant expression of miR-320 has also been reported in several cardiovascular diseases, such as coronary heart disease and heart failure [49, 52]. In particular, studies conducted in large cohorts reported miR-320b to be nominally downregulated in participants with a history of stroke and miR-320d to be associated with acute stroke [12, 53]. Our results add to this evidence, highlighting the miR-320 family as a key regulator of vascular function. In fact, besides the relation of miR-320 with vascular resistance and other traits of vascular health such as pulse wave velocity, systemic vascular resistance index and systolic blood pressure, our functional analysis revealed that miR-320 is involved in blood vessel development and morphogenesis, and angiogenesis. In particular, genes including *TGFBR1*, which encodes a receptor of the angiogenic growth factor *TGFB*, *ITGB1*, coding for integrin beta subunit, involved in cell-cell adhesions, and *RAP1A*, which signals for angiogenic growth of endothelial cells [54], were identified as miR-320 target genes. Notably, our results suggest that the relation of the miR-320 family with vascular function has even implications for brain health, as higher expression of this molecule was also related to increased WMH burden, a biomarker of cerebral small vessel disease. This notion was strengthened by our mediation analysis, which showed that total arterial compliance partially mediates the effect of miR-320 on WMH burden. Taken together, these findings suggest that an increase of miR-320 expression levels in the blood leads to a decrease in total arterial compliance, affecting the functioning of endothelial and vascular smooth muscle cells, as shown in previous studies [49–51]. The increased arterial stiffness can lead to impaired vascular function and blood-brain barrier disruption, ultimately contributing to the development of WMHs. Importantly, another study carried out by our group has provided suggestive evidence that higher miR-320 expression levels are also related to worse cognitive performance [55], indicating that the effect of miR-320 on (cerebro)vascular function might have implications for cognitive health. In contrast to our findings, Gao and colleagues found that exosomal miR-320e levels were negatively correlated with WMH load in a case-control study of 150 patients with cerebral small vessel disease and 80 control subjects [56]. This discrepancy, which could be due to the different study designs and populations and different biomaterials used to isolate microRNAs, highlights the need for further study of the miR-320 family in relation to brain and vascular health.

Interestingly, through our functional analysis, we identified a considerable overlap of biological processes regulated by the miR-320 family and miR-330-5p, despite these two microRNAs only sharing two target genes and their expression levels being related to worse and better arterial compliance, respectively. Notable overlapping pathways included tube morphogenesis, blood vessel development and morphogenesis, apoptotic signaling pathway, and cell-cell adhesion. In concordance with our finding of a relation with better arterial compliance, Zuo and colleagues reported that miR-330-5p might improve myocardial I/R injury and have anti-inflammatory activity by regulating the NLRP3 inflammasome [57].

In addition to miR-320 and miR-330-5p, our study showed that higher expression levels of miR-378 family were associated with higher arterial stiffness. The importance of this family for cardiovascular health is also highlighted by previous studies. Recently, Bu and colleagues revealed that overexpression of miR-378-3p promotes endothelial autophagy and leads to impaired endothelial function, by reducing endothelial nitric oxide synthase [58]. Another study found that miR-378c, derived from human coronary arteries, was downregulated in atherosclerotic plaques compared to normal controls. In line with that, it was also shown that miR-378c suppression enhances the phenotypic switch of vascular smooth muscle cells during atherosclerosis [59]. Notably, our functional analysis of confirmed miR-378 target genes showed, among others, an overrepresentation of biological processes related to telomere organization and maintenance. Several studies, including ours, have highlighted the relationship of telomere shortening with increased arterial stiffness, linking one of the major biomarkers of aging with a hallmark of vascular aging [60, 61]. Our findings indicate that the miR-378 family might have a role in linking these two aging-related processes, which was further supported by the age-dependent association of miR-378f and miR-378i with total arterial compliance index.

We identified an association of miR-6786-3p, miR-3605-3p and miR-6747-3p with total arterial compliance index and a strong association of miR-6786-3p with a large number of quantitative markers of cardiovascular function, highlighting its relevance as a common driver of cardiovascular health. Interestingly, we observed an age-dependent association of the three microRNAs with total arterial compliance index, as effect estimates were stronger in younger participants. To our knowledge, these microRNAs have not been previously reported to be implicated in vascular (dys)function, possibly because they were recently discovered, and thus were not included in the targeted microRNA quantification assays mostly employed by earlier studies, or because most studies thus far have focused on older subjects. The miR-eQTL analysis revealed that a strong genetic component influences the expression of miR-3605-3p, tagged by the SNP rs11554674. Specifically, our results showed that individuals carrying two copies of the effect allele had lower expression of miR-3605-3p, which based on our findings, will result in a worse total arterial compliance index. The rs11554674 was also identified to be nominally significant in a GWAS on pulse wave velocity, supporting the involvement of this SNP in arterial stiffness traits [62]. Interestingly, for miR-6747-3p we identified 76 common target genes with miR-330-5p, which suggests a coordinated repression effect, further supported by the common directionality of the association with arterial compliance. Among the biological processes significantly overrepresented in the functional annotation, it is worth highlighting the regulation of the insulin secretion pathway. It is well known that abnormal release of insulin contributes to arterial stiffness due to an uncontrolled activation of the renin-angiotensin-aldosterone system [63, 64].

Additionally, our clustering analysis showed that the expression levels of miR-192-5p were higher in participants with increased cardiac output. Impaired circulating miR-192-5p expression has been reported in several cardiovascular diseases such as hypertrophic cardiomyopathy and atrial fibrillation [65, 66]. Moreover, the “regulation of MAPK cascade” and “regulation of protein serine/threonine kinase activity” pathways arose from our functional analysis, conducted on confirmed miR-192-5p target genes. The MAPK signaling cascade controls many processes, from cell proliferation to cell differentiation and apoptosis. In the context of cardiovascular pathology, MAP kinases have been shown to be involved in cardiac hypertrophy, heart failure, and ischemia/reperfusion injury [67, 68].

Our study has several strengths. We were able to measure the association of microRNAs, isolated from peripheral whole blood, with a large panel of quantitative measurements of vascular health, in a population-based cohort. The hypothesis-free approach used in this study allowed us to investigate a large number of blood microRNAs, even identifying microRNAs for which little is still known. Moreover, we studied their effects on phenotypes related to cerebrovascular damage, leading to a comprehensive picture of the role of microRNA in vascular health. Finally, the integrative analysis of microRNA and gene expression data, obtained from the same set of samples, and the ensuing pathway analysis, contributed to disentangling the biological role of cardiovascular-related microRNAs.

Our study also has limitations. First, it was based on cross-sectional observational data, and the relations we observed between microRNA and quantitative measurements of vascular function cannot simply be interpreted as causal. While longitudinal studies or interventional trials could help establish a direct causal relationship, previous literature and our mediation analysis point towards a potential causal role of hub-microRNA miR-320 family on WMH burden through arterial compliance. Second, the integrative microRNA-gene expression analysis was conducted on candidate genes collected from three databases including only negative associations, so we may have missed some potential indirect interactions. Third, although our study is one of the biggest studies to date that investigates the relationship between microRNAs and vascular health, it might still have limited power to detect smaller effect sizes, due to insufficient sample size. Therefore, future studies are necessary to validate our findings, especially the ones which did not reach the conventional threshold of statistical significance at FDR < 0.05. Fourth, we evaluated indexed hemodynamic parameters, which is a standard approach in daily clinical practice and cardiovascular research. Components of the cardiovascular system, like the heart chambers and large arteries, are correlated to body size. Using hemodynamic parameters indexed to body surface area accounts to some extent for confounding due to factors such as sex, obesity and body size. However, we did not account for body composition and population-specific factors. While it is possible that these factors also influence the specific estimates of the parameters we investigated, we consider it unlikely that this would completely alter our findings. Moreover, the sensitivity analysis performed using non-indexed parameters revealed effect estimates comparable to those obtained using indexed-cardiovascular measurements, suggesting that body composition does not substantially influence the associations we identified. Lastly, the majority of participants in the Rhineland Study are of European descendent. Therefore, the generalizability of our findings to other populations may be limited.

## Conclusions

In conclusion, we showed that cardiovascular health is closely linked to the expression of specific circulating blood microRNAs, which primarily have an effect on arterial stiffness. Moreover, we found that the implications of this relation extend to brain health, as suggested by the association of miR-320 with WMH burden, mediated by arterial compliance. Additionally, we identified the biological processes in which the target genes are involved, such as blood vessel development and angiogenesis, thereby enhancing the understanding of the molecular mechanisms underlying vascular (dys)function. Overall, our findings highlight the crucial role of microRNAs as key regulators of vascular health, implicating them as potential targets for the development of novel preventive strategies against cardiovascular diseases in clinical settings.

### Electronic supplementary material

Below is the link to the electronic supplementary material.

Supplementary Material 1

Supplementary Material 2: Additional file 1: Fig. S1. Analysis of network topology. Fig. S2. Overlap among the analyzed datasets in the different analyses. Fig. S3. Relation of age with cardiovascular traits and eigen-microRNA vectors. Fig. S4. Relation between eigen-microRNA vectors and non-indexed cardiovascular traits. Fig. S5. Effect of age and sex on the relation between hub-microRNAs and total arterial compliance index. Fig. S6. Relation between hub-microRNAs and quantitative measurements of vascular function. Fig. S7. Hub-microRNAs and confirmed target genes.

Supplementary Material 3: Additional file 2: Table S1. microRNA-Module membership. Table S2. Association of eigen-microRNA vectors and cardiovascular traits. Table S3. Sensitivity analysis with non-indexed cardiovascular traits. Table S4. Mediation Analysis. Table S5. Interaction between age and hubMicroRNAs on cardiovascular traits. Table S6. Association between hubMicroRNAs and TACI according to age groups. Table S7. Interaction between sex and hubMicroRNAs on cardiovascular traits. Table S8. Association between hubMicroRNAs and TACI according to sex groups. Table S9. Association of hubmicroRNAs and cardiovascular traits. Table S10. Association hubmicroRNA-target genes. Table S11. Functional analysis - GO: BP. Table S12. miR-eQTL analysis.

## Data Availability

The data from the Rhineland Study are not publicly available due to data protection regulations. Access to data can be provided to scientists in accordance with the Rhineland Study’s Data Use and Access Policy. Requests for additional information or to access to the Rhineland Study’s datasets can be send to RS-DUAC@dzne.de.

## Abbreviations

CVD: Cardiovascular diseases
mRNA: Messenger RNA
WMH: White matter hyperintensity
MRI: Magnetic resonance imaging
SBP: Systolic blood pressure
DBP: Diastolic blood pressure
MAP: Mean arterial pressure
CVP: Central venous pressure
BSA: Body surface area
FLAIR: Fluid-attenuated inversion recovery
BMI: Body mass index
WGCNA: Weighted gene co-expression network analysis
TOM: Topological overlap matrix
FDR: False discovery rate
GO:BP: Gene Ontology: Biological Process
miR-eQTLs: MicroRNA expression quantitative trait loci
GWAS: Genome-wide association studies
MAPK: Mitogen-activated protein kinase
