# Supplementary material for: Peripheral whole blood microRNA expression in relation to vascular function: a population-based study
Source: J Transl Med. 2024 Jul 19;22:670. doi: 10.1186/s12967-024-05407-0 (PMC11264787; doi:10.1186/s12967-024-05407-0)
Supplement: Supplementary file 2 — Supplementary Material 2: Additional file 1: Fig. S1. Analysis of network topology. Fig. S2. Overlap among the analyzed datasets in the different analyses. Fig. S3. Relation of age with cardiovascular traits and eigen-microRNA vectors. Fig. S4. Relation between eigen-microRNA vectors and non-indexed cardiovascular traits. Fig. S5. Effect of age and sex on the relation between hub-microRNAs and total arterial compliance index. Fig. S6. Relation between hub-microRNAs and quantitative measurements of vascular function. Fig. S7. Hub-microRNAs and confirmed target genes. [file 12967_2024_5407_MOESM2_ESM.docx]

**Fig. S1. Analysis of network topology**


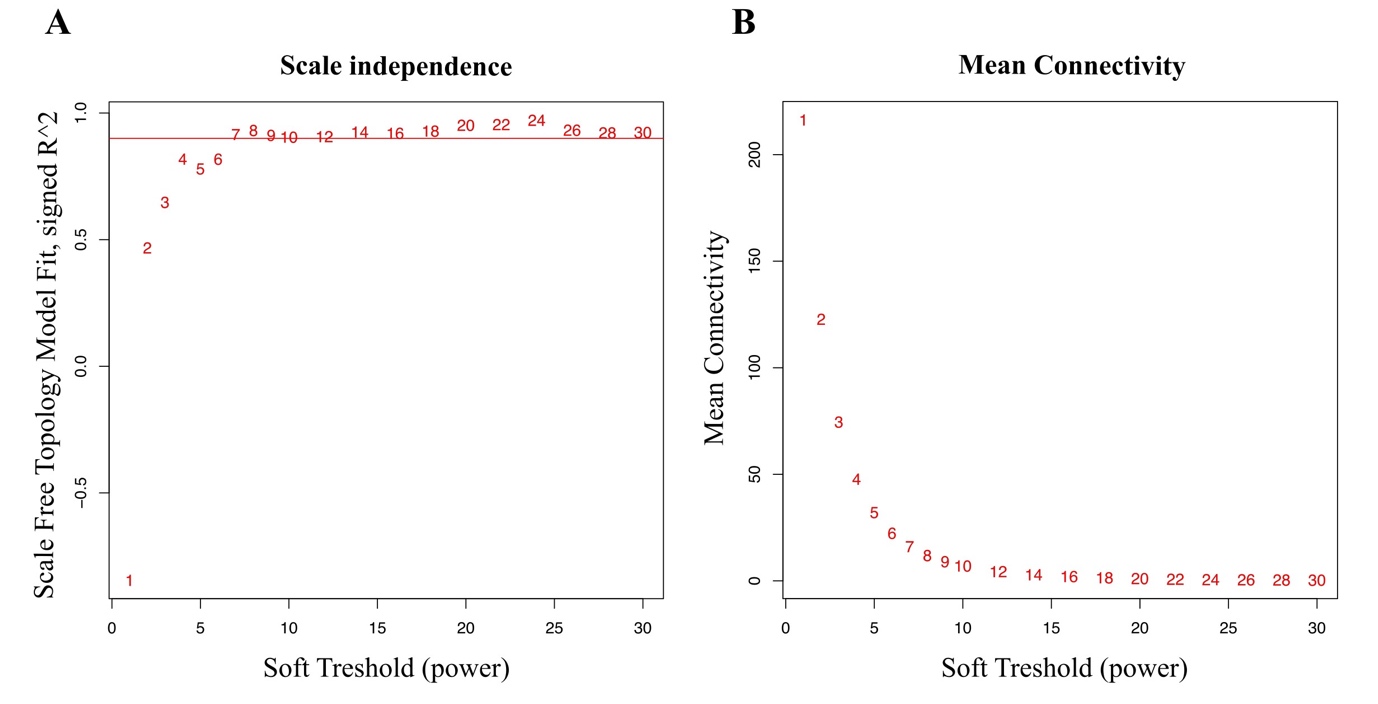


**(A)** Plot showing the scale-free fit index for different soft-threshold powers. **(B)** Plot showing the mean connectivity values for different soft-threshold powers.

**Fig. S2. Overlap among the analyzed datasets in the different analyses**


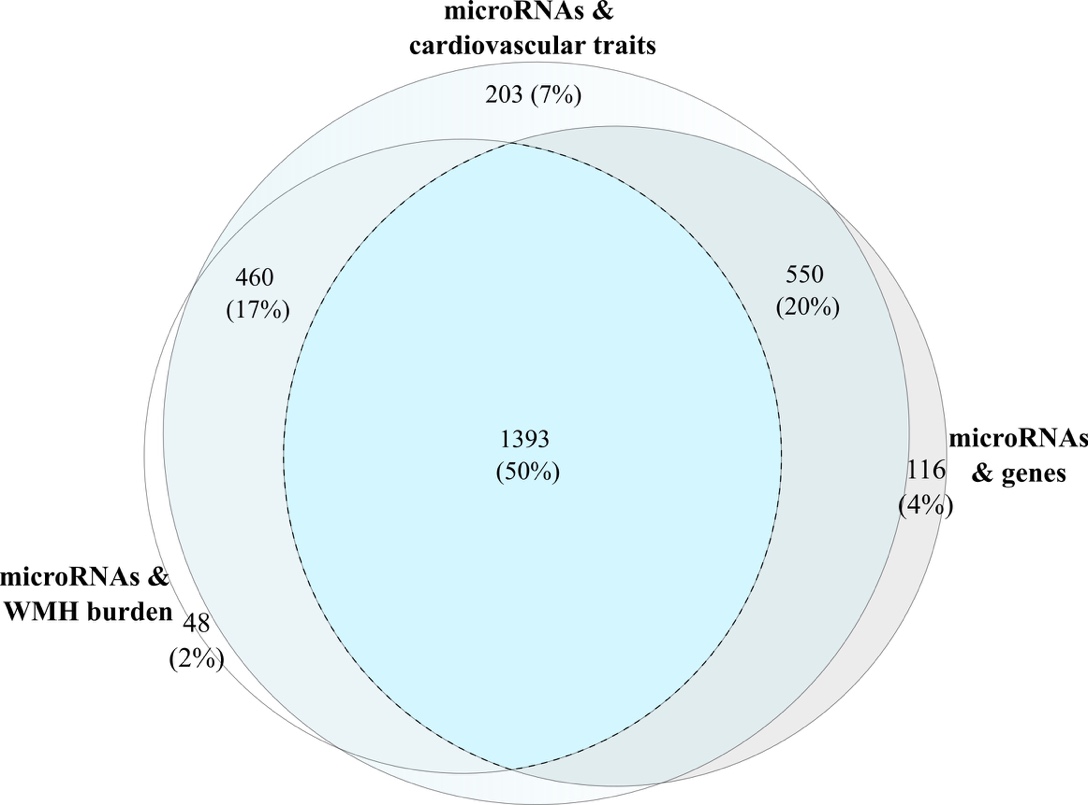


Venn diagram showing the intersection of participants included in the following analyses: 1) association of microRNA expression levels with cardiovascular traits; 2) association of microRNA expression levels with white matter hyperintensity burden; 3) association between the expression levels of microRNAs and the expression levels of candidate target genes. Abbreviation: WMH, white matter hyperintensity.

**Fig. S3. Relation of age with cardiovascular traits and eigen-microRNA vectors
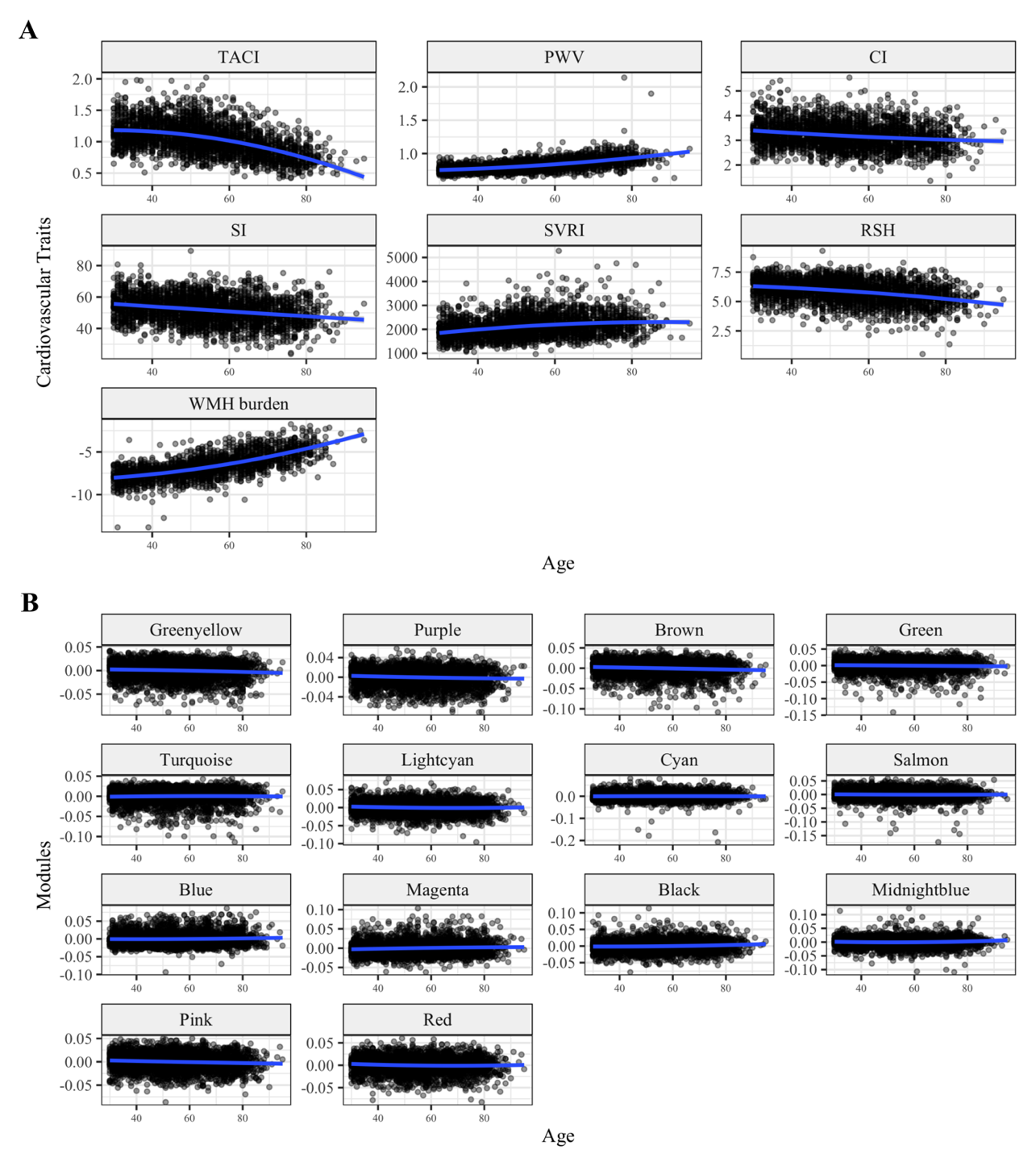
**

**(A)** Scatter plots showing the relationships between age and cardiovascular traits. **(B)** Scatter plots showing the relationships between age and module eigen-microRNA vectors. Abbreviations: CI, cardiac index; PWV, pulse wave velocity; RSH, reactive skin hyperemia; SI, stroke index; SVRI, systemic vascular resistance index; TACI, total arterial compliance index; WMH, white matter hyperintensity.

**Fig. S4. Relation between eigen-microRNA vectors and non-indexed cardiovascular traits**

**
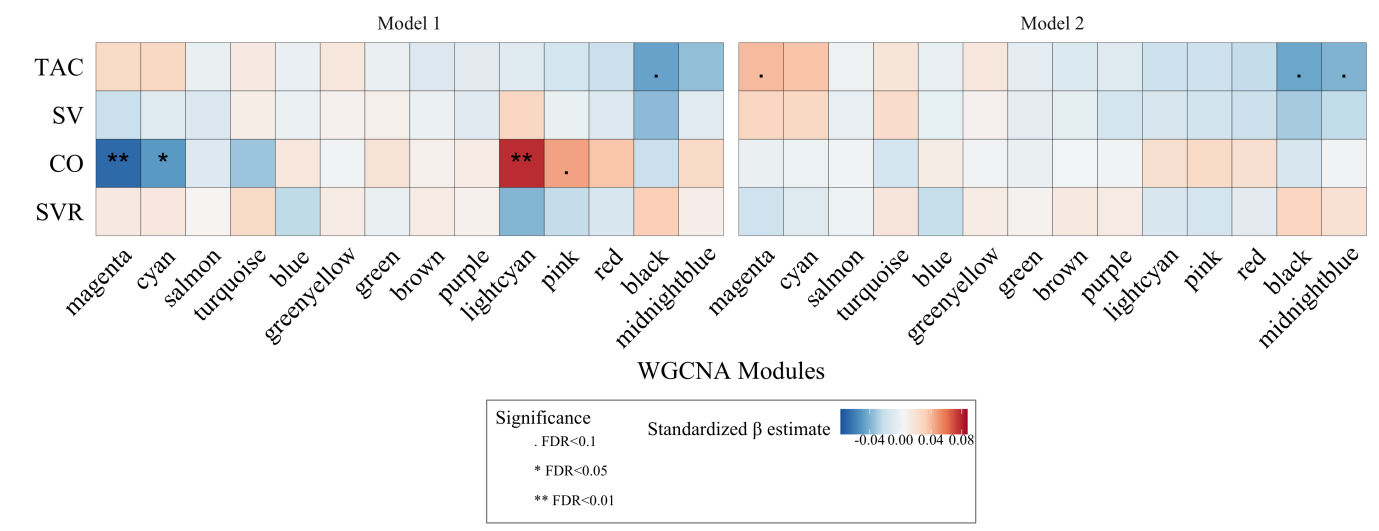
**

Heatmap showing the relation between module eigen-microRNA vectors (columns) and non-indexed cardiovascular traits (rows), assessed by multivariable linear models. *Model 1* is adjusted for age and sex. *Model 2* is further adjusted for body mass index. Abbreviations: CO, cardiac output; SV, stroke volume; SVR, systemic vascular resistance; TAC, total arterial compliance.

**Fig. S5. Effect of age and sex on the relation between hub-microRNAs and total arterial compliance index**

**
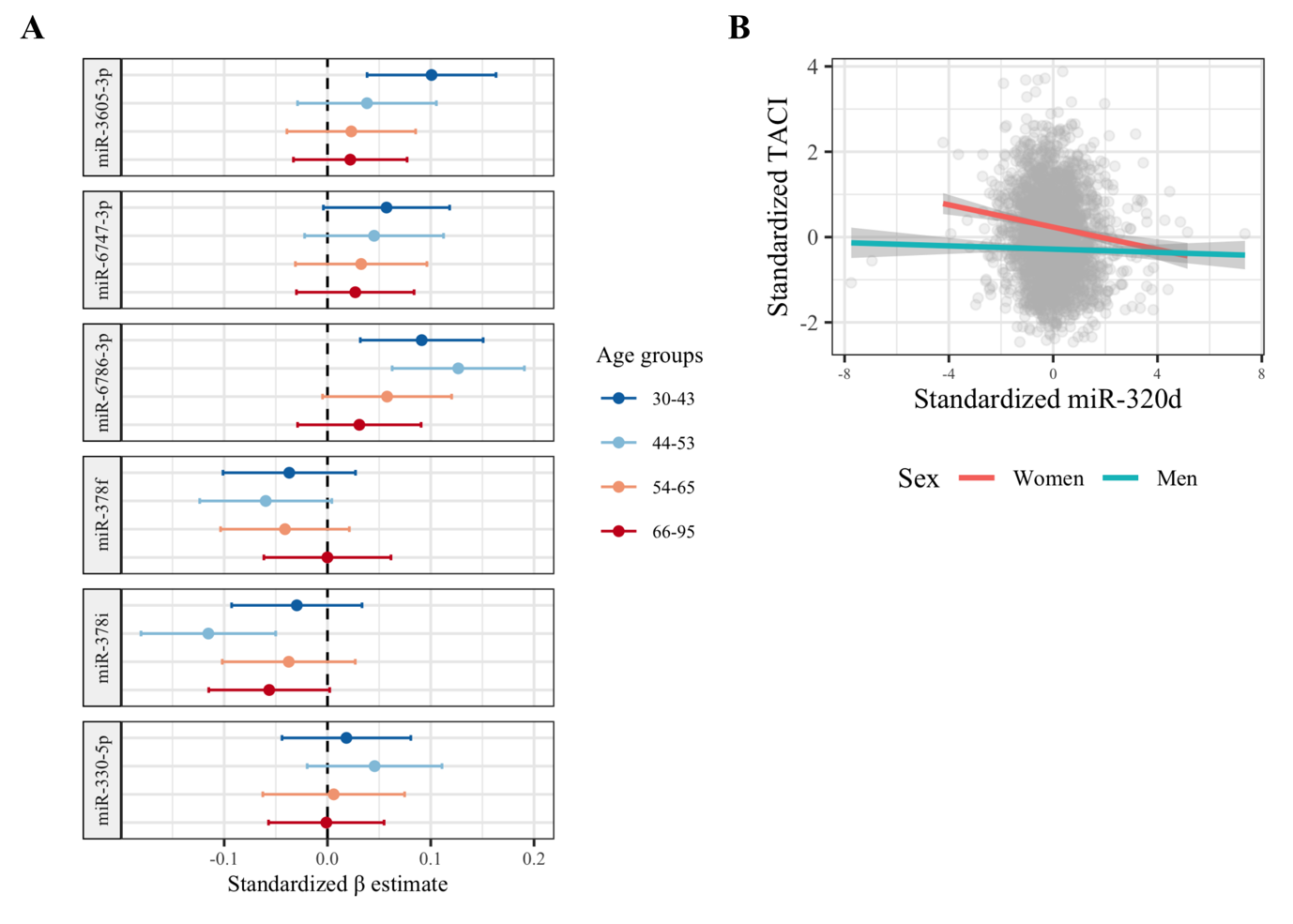
**

**(A)** Forest plot showing the age-stratified effect estimates for the association of candidate hub-microRNAs with total arterial compliance index. The age groups were defined based on the quartile function. **(B)** Interaction plot of miR-320d expression level and sex in relation to total arterial compliance index trait. Abbreviation: TACI, total arterial compliance index.

**Fig. S6. Relation between hub-microRNAs and quantitative measurements of vascular function**

**
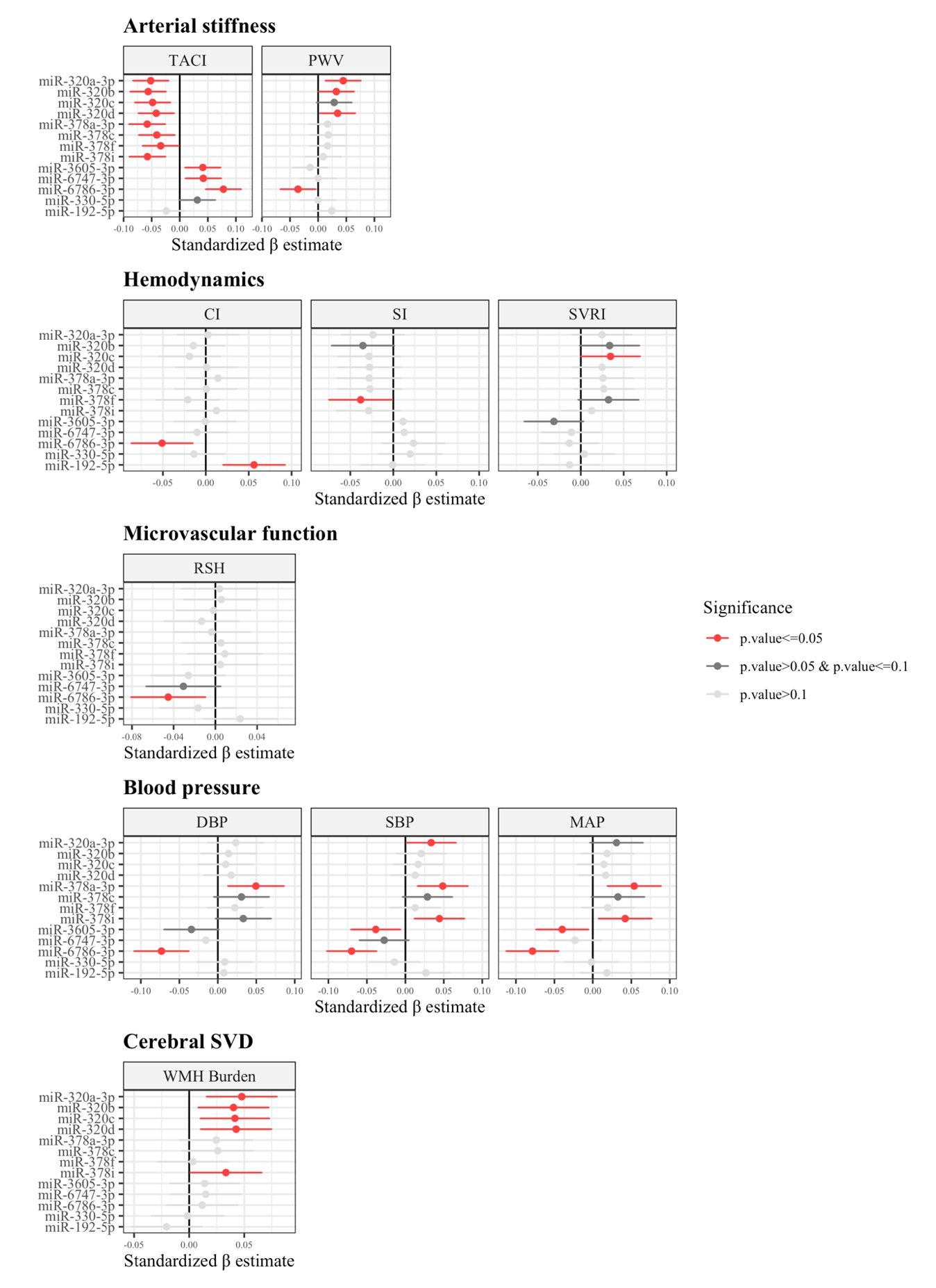
**

Forest plots showing the individual effect estimate of hub-microRNAs on vascular traits. The standardized β estimates are obtained through the following linear regression model: Trait_x_ ~ intercept + hub-microRNA_i_ + age + sex. The statistical significance level is set at p-value<0.05. Abbreviations: CI, cardiac index; DBP, diastolic blood pressure; MAP, mean arterial pressure; PWV, pulse wave velocity; RSH, reactive skin hyperemia; SBP, systolic blood pressure; SI, stroke index; SVD, small vessel disease; SVRI, systemic vascular resistance index; TACI, total arterial compliance index; WMH, white matter hyperintensity.

**Fig. S7. Hub-microRNAs and confirmed target genes**

Network plot showing the 13 hub-microRNAs and the corresponding confirmed target genes. Each dot represents a target gene, confirmed through the integration analysis of microRNA and gene expression data in the Rhineland Study.
