# Supplementary material for: Peripheral whole blood microRNA expression in relation to vascular function: a population-based study
Source: J Transl Med. 2024 Jul 19;22:670. doi: 10.1186/s12967-024-05407-0 (PMC11264787; doi:10.1186/s12967-024-05407-0)
Supplement: Supplementary file 3 — Supplementary Material 3: Additional file 2: Table S1. microRNA-Module membership. Table S2. Association of eigen-microRNA vectors and cardiovascular traits. Table S3. Sensitivity analysis with non-indexed cardiovascular traits. Table S4. Mediation Analysis. Table S5. Interaction between age and hubMicroRNAs on cardiovascular traits. Table S6. Association between hubMicroRNAs and TACI according to age groups. Table S7. Interaction between sex and hubMicroRNAs on cardiovascular traits. Table S8. Association between hubMicroRNAs and TACI according to sex groups. Table S9. Association of hubmicroRNAs and cardiovascular traits. Table S10. Association hubmicroRNA-target genes. Table S11. Functional analysis - GO: BP. Table S12. miR-eQTL analysis. [file 12967_2024_5407_MOESM3_ESM.pdf]

Response to the comments about the submitted paper 'Peripheral whole blood microRNA expression in relation to vascular function: a population-based study' (JTRM-D-24-01680)

We thank the reviewers for their helpful comments and recommendations. We have adapted our manuscript accordingly. Most important changes include a better explanation of the rationale behind including results that are borderline with the conventional threshold of statistical significance (0.05), creating a specific subsection in the methods and results sections for hub-microRNAs, clearly stating pre-existing cardiovascular conditions and exclusion criteria for participants, exploring the relationship of age with microRNAs and with cardiovascular measurements, and expanding the information about vascular measurements.

We have indicated modifications done in the manuscript by highlighting them in red and specifying the corresponding line in the explanation below.

Here, we address each comment directly:

- **Answer to Reviewer 1**;
- **Answer to Reviewer 2**;
- **Answer to Reviewer 3**;
- **Answer to Reviewer 4**;
- **Answer to Reviewer 5**.

Answer to Reviewer 1

General Comments

I've reviewed the attached manuscript titled "Peripheral whole blood microRNA expression in relation to vascular function: a population-based study." This comprehensive study explores the associations between whole blood microRNAs and various measures of vascular function in a population-based cohort. It identifies microRNA modules associated with arterial compliance, cardiac index, and white matter hyperintensity burden, offering insights into their potential roles in vascular health and disease. The study employs advanced statistical and bioinformatics analyses to elucidate the biological functions of these microRNAs, contributing significantly to our understanding of cardiovascular disease mechanisms and suggesting potential targets for prevention and therapy.

R1.1: *Title, Abstract, Keywords*

The manuscript titled "Peripheral whole blood microRNA expression in relation to vascular function: a population- based study" explores the relationship between microRNA expression in whole blood and vascular health indicators. The abstract effectively summarizes the study's methodology, significant findings, and their implications for understanding vascular function and potential cardiovascular disease interventions. However, critical and detailed feedback would focus on enhancing clarity around the specific vascular health measures examined and perhaps providing a more direct link between the identified microRNAs and potential clinical applications. The keywords are well-chosen, covering all relevant areas of the study, but could be expanded to include specific vascular health measures studied, such as "arterial stiffness" or "white matter hyperintensity," to improve searchability and relevance.

Answer: According to the comment, we have listed the vascular measurements used in the study. We have reported the edited section below:

Line 56: Through linear regression models, we investigated the association between each module's expression and quantitative markers of vascular health, including pulse wave velocity, total arterial compliance index, cardiac index, stroke index, systemic vascular resistance index, reactive skin hyperemia and white matter hyperintensity burden.

Additionally, we have included "white matter hyperintensity" as an additional keyword. As per the guidelines of the BMC Journal of Translational Medicine, we are limited to 10 keywords, so we're unable to add any more terms.

R1.2: *Background Section*
The background section of the manuscript provides a comprehensive overview of the current understanding of the role of microRNAs in cardiovascular diseases (CVD) and outlines the research objectives of the study. Here are several critical, detailed, and specific comments and suggestions to enhance the clarity, depth, and scientific rigor of the background:
R1.2.1: *Literature Review Completeness and Currency:*
The authors have done a commendable job summarizing the role of microRNAs in vascular biology and CVD. However, it would be beneficial to include more recent studies to ensure the literature review is up-to-date, reflecting the latest advancements in the field. The inclusion of studies up until the current year would strengthen the argument and provide a more solid foundation for the study's objectives.

Answer: We have replaced one reference and included an additional one:
- Reference "Quiat D, Olson EN. MicroRNAs in cardiovascular disease: from pathogenesis to prevention and treatment. J Clin Invest. 2013" has been replaced by "Zhou SS, Jin JP, Wang JQ, Zhang ZG, Freedman JH, Zheng Y, et al. miRNAS in cardiovascular diseases: potential biomarkers, therapeutic targets and challenges. Acta Pharmacol Sin. 2018" [#8];
- "Zhang J, Starkuviene V, Erfle H, Wang Z, Gunkel M, Zeng Z, et al. High-content analysis of microRNAs involved in the phenotype regulation of vascular smooth muscle cells. Sci Rep. 2022" has been included [#10].

R1.2.2: *Clarification of Study Rationale:*
While the authors have outlined the importance of microRNAs in CVD, the transition from the general role of microRNAs to the specific focus of the study (circulating microRNAs and vascular function) could be made clearer. A brief explanation of why circulating microRNAs, in particular, are of interest would help to strengthen the rationale for the study.

Answer: To address this comment, we have added a short sentence which highlights the rationale behind the use of microRNAs in the blood:

Line 101: In addition, aberrant expression levels of several circulating microRNAs have been observed in CVDs, such as coronary syndromes, heart failure and stroke [12-14]. Circulating microRNAs have garnered increasing attention as promising diagnostic and

prognostic biomarkers for CVDs due to their stability in body fluids even under harsh conditions, easy accessibility, and distinctive diseases-related patterns [15].

R1.2.3: *Specific Examples and Mechanisms:*
The manuscript mentions microRNAs such as miR-145 and miR-126 but does not delve deeply into the mechanisms through which these microRNAs influence vascular function or contribute to CVD. Providing a more detailed explanation of a few key mechanisms would help readers understand the complexity and significance of microRNA functions in vascular biology.

Answer: We have briefly explained in which biological mechanisms these 2 microRNAs are involved in the following section:

Line 97: In vitro and animal studies have identified microRNAs implicated in the regulation of endothelial cell homeostasis and angiogenesis, or in the promotion of vascular dysfunction [7, 8]. Examples include miR-145, which is implicated in the regulation of vascular smooth muscle cell proliferation [9, 10], and miR-126 which enhances angiogenesis [11].
However, our aim in including this section was to provide just a few examples of microRNAs involved in vascular function, so we would like to keep this section relatively short.

R1.2.4: *Discussion on Methodological Limitations of Previous Studies:*
The authors mention limitations in previous human studies, including a reliance on in silico analyses and a lack of functional validation. Expanding on how these limitations could have impacted the findings of previous research and how this study aims to overcome them would add depth to the background.

Answer: We have adjusted the section in the following way:

Line 110: The majority of existing research derives from animal models [16] or relatively small observational studies, with a primary focus on specific microRNAs in relation to single vascular traits [17, 18], limiting the understanding of their impact on overall vascular health. Moreover, the limited number of population-based studies employing hypothesis-free approaches has predominantly concentrated on clinical endpoints [12, 13] rather than endophenotypes, which may limit the understanding of disease mechanisms. Additionally, in human studies, the biological functions of microRNAs are usually investigated only in silico by querying microRNA target prediction tools, without further functional validation due to the lack of gene expression data [19].

We have included in the manuscript the approaches we will use to overcome these limitations. Specifically, we will use a comprehensive panel of vascular measurements and will leverage gene expression data from the Rhineland Study to identify biological mechanisms in which the candidate microRNAs are involved (Line 119).

R1.2.5: *Justification for the Three-Stepped Approach:*
The study's three-stepped approach is briefly outlined but lacks a detailed justification for each step. Explaining how each step builds upon the previous one

and contributes uniquely to the understanding of microRNAs in vascular function would help to highlight the study's innovative aspects.

Answer: We employed a three-stepped approach to address the limitations of previous studies, which primarily investigated the association of microRNAs with individual vascular traits or clinical endpoints, and they conducted functional analyses only in silico. Specifically, we used a comprehensive panel of vascular measurements, including arterial stiffness traits, hemodynamics measurements and white matter hyperintensity to gain an understanding of overall vascular health. Moreover, we used gene expression data to investigate the functional biological role of candidate microRNAs. Finally, we performed a miR-eQTL analysis to investigate whether specific genetic variants affect the expression level of candidate microRNAs (Line 120).

R1.2.6: *Clarify Novelty and Expected Contributions:*
While the study aims are mentioned, a clearer statement regarding the expected contributions of this research to the field of cardiovascular biology and medicine would be beneficial. Specifically, how the findings might influence future research directions, clinical practice, or both should be articulated.

Answer: We have a section related to how our findings might influence future researches and clinical practice in the discussion part, specifically in the conclusion section.
We think that addressing this aspect in the conclusion part of the manuscript provides a more suitable context (Line 714).

R1.2.7: *References and Citations:*
Ensure that all references are current and accurately reflect the statements made. In some instances, broader claims about microRNAs regulating a significant portion of protein-coding genes are made; these claims should be supported by the most recent and robust evidence. Additionally, checking for any seminal works that may have been missed would improve the literature review's comprehensiveness.

Answer: Thank you for the comment. Based on this feedback and the feedback # **R1.2.1**, we have updated the reference list, by including the following two papers:

- Zhou SS, Jin JP, Wang JQ, Zhang ZG, Freedman JH, Zheng Y, et al. miRNAS in cardiovascular diseases: potential biomarkers, therapeutic targets and challenges. Acta Pharmacol Sin. 2018"[#8];
- "Zhang J, Starkuviene V, Erfle H, Wang Z, Gunkel M, Zeng Z, et al. High-content analysis of microRNAs involved in the phenotype regulation of vascular smooth muscle cells. Sci Rep. 2022" [#10].

We tried to choose references that are recent but also highly relevant to our topic. Therefore, the choice of including the article https://doi.org/10.1016/j.cell.2009.01.002, which, although not recent, is one of the top articles explaining the "microRNA target recognition" topic.

R1.2.8: *Addressing Potential Biases and Limitations:*

A brief discussion of the potential biases and limitations inherent in the study of microRNAs related to CVD, including those related to study design, sample selection, and the generalizability of findings, would provide a more balanced and critical perspective.

Answer: We have considered your feedback regarding the inclusion of a discussion on potential biases and limitations in the introduction section. However, we believe that addressing these aspects in the limitation section of the discussion provides a more appropriate context.

In conclusion, the background section lays a solid foundation for the study but would benefit from expansions in several areas to enhance its clarity, depth, and relevance. Addressing these comments will strengthen the manuscript's argument for the significance and novelty of the proposed research.

R1.3: *Methods Section*
The "Methods" section of the manuscript provides a detailed description of the study design, participant selection, data collection, and analytical strategies employed. This comprehensive presentation is commendable for its attempt to ensure transparency and reproducibility. However, several areas could benefit from further clarification, refinement, or consideration to align with the highest standards of methodological rigor expected by leading scientific journals. Here are specific, critical, and detailed comments and suggestions.

R1.3.1: *Data Availability and Ethical Considerations*
The explanation of data availability due to data protection regulations is appreciated. However, it could be beneficial to provide more detail on the nature of the Data Use and Access Policy, especially regarding the criteria or prerequisites for data access. This would offer clarity for future researchers interested in collaborating or validating the study findings.

Answer: We recognize that providing details about the criteria or prerequisites for data access may be important for potential collaborators. However, we believe that including this specific information in the manuscript might exceed its scope. Instead, we have provided the email address of the Data Use and Access Committee (DUAC) for researchers to contact us and request additional information.

R1.3.2: *Study Population and Selection Bias*
The selection of participants from the Rhineland Study is well-described. However, the manuscript could benefit from a discussion on potential selection biases and their implications for the generalizability of the results. Given the geographical and demographic specificity of the cohort, it is crucial to address how these factors might influence the findings and their applicability to other populations.

Answer: Thank you for the comment. We have now added a comment on this in the discussion, including this as a possible limitation of our study.

Line 703: Lastly, the majority of participants in the Rhineland Study are of European descendent. Therefore, the generalizability of our findings to other populations may be limited.

R1.3.3: *Vascular Function Measurements*
The detailed description of vascular function measurements is a strong aspect of the methodology. Nonetheless, for completeness, the authors should consider including the validation status of the devices and techniques used (e.g., Omron 705 IT, CardioScreen 2000) against gold-standard measures. This information would add credibility to the measurement accuracy and reliability of the data collected.

Answer: Thank you for the feedback. We have included in the manuscript more detailed information on the devices and techniques used, and corresponding references:

Line 170: Systolic blood pressure (SBP) and diastolic blood pressure (DBP) were measured in a sitting position, using an oscillometric blood pressure device (Omron 705 IT) [22, 23]. The measurements were performed thrice, separated by ten minutes, by experienced study technicians while participants were sitting in a resting chair in a quiet environment. Cuff size was determined by measuring participants' arm circumference in the middle of the upper arm between the acromion and olecranon on the right arm of the participant sitting in the measuring position. Measurements were preferably performed in the right arm. In cases where the measurements were not possible on the right arm, the left arm was used. The measured arm was always placed in a resting position at heart level, with the palms facing upward, the shoulders in a horizontal position, and both legs resting on the ground.

Line 183: Impedance cardiography was performed with the CardioScreen 2000 device (Medis, Germany), by experienced study technicians, in a temperature-controlled room. Before the examinations, the participants were placed in a supine position and allowed to rest for five minutes. Electrodes as well as arm, ankle and thigh cuffs were placed as per the device manufacturer's recommendations [25]. All hemodynamic measurements were calculated by in-developed software, based on simultaneously registered electrocardiography signals and blood pressures with 2-minute intervals.

R1.3.4: *MicroRNA and Gene Expression Profiling*
The sequencing and profiling methods are thoroughly detailed, which is essential for the reproducibility of the results. It would be beneficial to include a brief rationale for the choice of sequencing platforms and library preparation kits, as well as any limitations or biases these choices might introduce. Additionally, detailing the criteria for excluding low-quality reads and the thresholds for inclusion in the analysis would strengthen this section.

Answer: We have added the following detail accordingly to the comment:

Line 248: The quality of the sequencing reads was evaluated through FastQC v0.11.9 software. Sequencing adapters, low-quality score reads (i.e., reads with the average

quality per 4-base wide below 15) and reads shorter than 18 base pairs were discarded using Trimmomatic v0.39 software.
The rationale for choosing Illumina sequencing platforms and PAXgene Blood miRNA kit is their extensive use within the research community for next-generation sequencing.

R1.3.5: *Statistical Analysis and Model Adjustments*
The statistical methods employed for data analysis, including WGCNA, linear regression models, and miR- eQTL analysis, are appropriately detailed. However, the rationale behind choosing specific models and methods (e.g., threshold power of 7 for WGCNA, adjustment variables in regression models) could be further elaborated. Clarification on how these choices were informed by preliminary analyses or existing literature would add depth to the methodological justification.
It is also recommended to discuss the potential impact of unmeasured confounding factors on the study findings and how these were addressed or mitigated.

Answer: To address this question, we have run additional sensitivity analyses. We have controlled for smoking and genetics predispositions, as suggested by Reviewer 3, question number 2. For more details, please refer to the question number 2 of reviewer 3.

R1.3.6: *Genome-wide miR-eQTL Analysis*
The miR-eQTL analysis section is a critical component of the methodology, offering insights into the genetic regulation of microRNA expression. The decision criteria for defining cis- and trans-SNPs should be further justified, particularly the choice of a 1 Mb threshold for cis-SNPs. Additionally, discussing the potential for population stratification effects in the analysis and how these were accounted for would be valuable.

Answer: Regarding the criteria for defining cis- and trans- SNPs, we have used the same window reported in a previous paper that conducted miR-eQTL analysis in 280 microRNAs (Huan T, Rong J, Liu C, Zhang X, Tanriverdi K, Joehanes R, et al. Genome-wide identification of microRNA expression quantitative trait loci. Nat Commun. 2015;6:6601). We have now referenced the paper in the manuscript.
Regarding population stratification in the miR-eQTL analysis, to account for it we have adjusted the model for the first 10 principal components. We report the section where we indicate the model used for miR-eQTL analysis:

Line 394: Specifically, we first adjusted microRNA expression levels for age, sex, the first 10 genetic principal components and batch effects, and extracted the residuals. Subsequently, linear regression analysis was used to assess the relation between each SNP (independent variable) and these hub-microRNA residuals (dependent variable).

R1.3.7: *Overrepresentation and Functional Analysis*
The approach to mapping genes regulated by hub-microRNAs and the subsequent functional analysis is a significant strength of the study. However, elaborating on the criteria for selecting databases for experimentally validated and predicted target genes would enhance the transparency of the methodological approach.

Furthermore, a discussion on the limitations of overrepresentation analyses, including potential biases introduced by database completeness and the interpretation of semantically similar GO terms, would provide a more nuanced understanding of the findings.

Answer: Thank you for pointing out this. We have adjusted the section regarding online databases for identifying target genes accordingly:

Line 367: First, we collected data on experimentally validated target genes and predicted target genes using the R package multiMir v1.12.0. Specifically, we queried MirTarBase [40] and TargetScan [41], widely recognized online databases for microRNA target gene prediction, and miRDB [42], a database providing experimentally validated microRNA-target interactions.

R1.3.8: *Considerations for Reproducibility and Transparency*
The manuscript would benefit from a more detailed description of the software versions and settings used for statistical analyses and data processing. Providing access to the custom scripts or workflows used in the analysis, possibly through a public repository, would significantly enhance the reproducibility and transparency of the research.

We agree that stating the software versions and settings used for statistical analyses is crucial for reproducibility and transparency. Therefore, we consistently provided information on the version of R packages or other software utilized in the study, along with specific parameters that we consider important for readers intending to replicate the analyses. For instance, this included details such as the threshold power of 7 and cutHeight of 0.2 for WGCNA analysis, or the low similarity threshold of 0.5 for rrvgo analysis.

Conclusion

Overall, the methodology section of the manuscript is thorough and detailed, laying a solid foundation for the study. Addressing the comments and suggestions provided here will further strengthen the methodological rigor, clarity, and transparency of the research, aligning it with the high standards expected by top-tier scientific journals.

R1.4: *Results Section*
The "Results" section of your manuscript provides a thorough and detailed account of the findings from a comprehensive study on the role of microRNAs in vascular function and their potential links to cardiovascular health. The structured presentation, encompassing participant characteristics, microRNA co-expression network analysis, identification of hub-microRNAs, their associations with vascular functions, and the miR-eQTL analysis, is commendable. However, to meet the stringent requirements of high-impact journals, several aspects could be enhanced or clarified further. Here are some critical, detailed, and specific comments and suggestions:

R1.4.1: *Clarity and Precision in Reporting Results*
Ensure that all figures and tables referenced are clearly described and their relevance is explicitly stated in the text. For example, the manuscript mentions Figures 1, 2, 3, 4, 5, and Tables 1, 2, 3 but does not provide a clear narrative that guides the reader through these

visual and tabular presentations. A more detailed description of each figure/table's key findings within the text would enhance readability and understanding.

Answer: We understand that a clearly description of figures and tables is crucial for reader comprehension. However, to improve the manuscript's readability, especially given the abundance of results presented in the manuscript, we have opted to include detailed descriptions of figures and tables in their legends.

R1.4.2: *Statistical Analysis and Interpretation*
The manuscript does well to apply rigorous statistical methods. However, the interpretation of statistical significance and the biological relevance of the findings could be expanded. For instance, while FDR-adjusted p-values are reported, a discussion on the effect sizes, confidence intervals, and their implications for vascular health and disease would add depth to the analysis. Furthermore, it's vital to discuss the potential clinical relevance of these associations, even if preliminary, to contextualize the findings within the broader field of cardiovascular research.

Answer: Thank you for the comment. Similarly to the previous suggestion, to improve the manuscript's readability, we have opted to report the effect sizes of only significant results. More detailed information (e.g., confidence intervals, F statistics…) is available in the tables or supplementary tables, where the reader can find the summary statistics of all the analyses conducted. Additionally, we have considered your feedback regarding the contextualization of the findings within the broader field of cardiovascular research. However, we feel that this aspect is better addressed in the discussion part.

R1.4.3: *Integration of miR-eQTL Analysis*
The miR-eQTL analysis provides valuable insights into the genetic regulation of microRNA expression. However, the discussion on how these findings integrate with the observed associations between microRNA expression levels, vascular function, and cardiovascular health is somewhat limited. Expanding on the potential mechanisms through which identified eQTLs might influence microRNA function and subsequent vascular outcomes could provide a more comprehensive understanding of the study's implications.

Answer: In the result section, we have reported only the actual results of the miR-eQTL analysis. However, we have elaborated on potential mechanisms through which identified eQTLs might influence microRNA expression levels and subsequent vascular traits in the discussion section.

R1.4.4: *Hub-microRNAs and Functional Analysis*
The identification of hub-microRNAs and the subsequent functional analysis represent key strengths of the manuscript. Nonetheless, the presentation and discussion of these results could be improved by more explicitly linking the identified microRNAs and their target genes to known pathways and mechanisms involved in cardiovascular disease. This would not only validate the findings but also suggest potential therapeutic targets or biomarkers for further investigation.

Answer: Similarly to the previous comment, in the result section, we have included only the pathways that reached statistical significance at p.adjusted<0.05 and were related to vascular function. We then elaborated further on the link between the candidate microRNAs, target genes and enriched pathways in the discussion section.

R1.4.5: *Sensitivity Analysis and Confounding Factors*
The manuscript mentions conducting sensitivity analyses, particularly regarding the effect of BMI on the associations observed. While this is a good practice, elaborating on other potential confounding factors that were considered (or could not be adjusted for) and how they might impact the study's findings would provide a more nuanced interpretation of the results.

Answer: Based on this comment and the feedback from reviewer 3, question 2, we conducted additional exploratory analyses. In these analyses, we controlled the association between microRNAs and vascular traits for smoking and genetic predispositions. For more details, please refer to the question number 2 of reviewer 3.

R1.4.6: *Generalizability and Population-Specific Findings*
The study population's characteristics are well-detailed, yet the manuscript could further address the generalizability of the findings to other populations. Given the cohort's demographic and geographical specificity, discussing the limitations regarding the extrapolation of results to different populations or ethnicities would be valuable.

Answer: Following your feedback, we have added the generalizability of the findings as a limitation of our study since the participants of the Rhineland Study are mainly European descendant:

Line 703: Lastly, the majority of participants in the Rhineland Study are of European descendent. Therefore, the generalizability of our findings to other populations may be limited.

R1.4.7: *Limitations*

While implicit in various sections, a concise, dedicated discussion on the study's limitations, including aspects of the methodology, analysis, and interpretation, would enhance the manuscript's rigor. This includes acknowledging the observational nature of the study and its implications for causal inference.

Answer: We agree that a limitation section is necessary for the manuscript's rigor. However, we opted to have this section in the discussion (Line 679).

Conclusion
Overall, the "Results" section presents a wealth of detailed findings that significantly contribute to understanding microRNAs' role in vascular function and cardiovascular health. Addressing the above comments will enhance the clarity, depth, and impact of the manuscript, ensuring it meets the high standards of the targeted prestigious journals.

More Comments on Results Section

This analysis presents a detailed exploration of the relationship between microRNA expression patterns, vascular function, and cardiovascular health implications. The use of sophisticated methodologies, such as weighted microRNA co-expression network analysis (WGCNA) and genome-wide miR-eQTL analysis, provides a robust framework for elucidating these complex biological relationships. Herein, I delineate several advanced, comprehensive, and critical remarks, alongside recommendations for future research directions and methodological enhancements.

1. *Data Validation*

The manuscript provides an extensive validation of microRNA-target gene associations using an integrative approach that combines microRNA and gene expression data. However, external validation using independent cohorts or experimental validation in cell or animal models would further strengthen the causal inference between identified microRNA expressions and vascular functions.

2. *Data Integration*

The integration of microRNA expression profiles with gene expression data and genome-wide association signals exemplifies a commendable multi-omics approach. This holistic strategy enhances the understanding of the molecular underpinnings of cardiovascular health. Future studies might benefit from incorporating additional omics layers, such as metabolomics or proteomics, to provide a more comprehensive view of the regulatory networks involved.

3. *Strengths and Significance*

A significant strength of this study is its population-based design, which encompasses a broad spectrum of vascular function markers and employs advanced bioinformatic tools for network analysis and eQTL mapping. This allows for a nuanced exploration of microRNA roles in cardiovascular health, offering potential avenues for therapeutic interventions and biomarker development.

4. *Novelty*

The identification of hub-microRNAs within key modules related to vascular functions and their association with specific cardiovascular traits represents a novel contribution to the field. Particularly, the discovery of genetic loci influencing microRNA expression provides new insights into the genetic architecture of vascular health.

5. *Significance*

This research underscores the critical role of microRNAs in mediating vascular health, highlighting their potential as targets for therapeutic intervention and as biomarkers for cardiovascular disease risk assessment. Its significance lies in bridging the gap between genetic predisposition, microRNA regulation, and vascular disease manifestation.

6. *Drawbacks*

The study's reliance on observational data from a single cohort limits its ability to establish causality and raises concerns about the generalizability of the findings across different populations. Furthermore, the absence of functional validation of microRNA-target interactions in vitro or in vivo represents a limitation in confirming the biological relevance of these associations.

7. *Best Solution*

To address these drawbacks, future studies should aim to replicate the findings in diverse and larger cohorts to validate the generalizability of the observed associations. Additionally, conducting functional experiments would provide mechanistic insights into how specific microRNAs influence vascular functions and contribute to cardiovascular

disease.

8. *Reproducibility*

The detailed methodological description and the use of publicly available databases and standardized bioinformatics tools enhance the reproducibility of the study. Sharing data and code through open-access repositories could further improve reproducibility and facilitate future research.

9. *Potentially Further and Futuristic Researches*

Expanding this research to explore the longitudinal changes in microRNA expression and their impact on vascular health outcomes would be valuable. Additionally, investigating the interaction between lifestyle factors, microRNA expression, and genetic predisposition could offer a holistic understanding of cardiovascular disease etiology.

10. *Conclusions*

The findings from this study illuminate the complex interplay between microRNAs, gene expression, and genetic factors in modulating vascular functions, offering novel insights into cardiovascular disease mechanisms. These results lay the groundwork for further exploration into microRNA-based therapeutic strategies and biomarker development for cardiovascular health.

11. *Recommendations*

Future studies should focus on external validation, functional verification of microRNA-target gene interactions, and exploration of additional omics data to enrich the understanding of cardiovascular health regulation.

Engaging in interdisciplinary collaborations could accelerate the translation of these findings into clinical applications and personalized medicine approaches.

R1.5: *Discussion Section*

The "Discussion" section provides an insightful synthesis of the findings, contextualizing the impact of microRNA expression levels on vascular health and brain function within a large population-based cohort. The identification of specific microRNA families, such as miR-320 and miR-378, and their association with vascular dysfunction, offers significant contributions to the understanding of cardiovascular diseases' molecular mechanisms. Here are several critical, detailed, and specific comments to enhance the manuscript further.

R1.5.1: *Causality and Observational Nature of the Study:*

The discussion adeptly addresses the limitations inherent in inferring causality from observational data. While the authors suggest potential causal roles for certain microRNAs based on mediation analysis, it would be beneficial to emphasize more strongly the need for longitudinal studies or interventional trials to establish direct causal relationships. Additionally, discussing methods such as Mendelian randomization could offer avenues for future research to validate these findings causally.

Answer: Thank you for the comment. Based on your suggestion, we have slightly adapted the section in the following manner:

Line 679: Our study also has limitations. First, it was based on cross-sectional observational data, and the relations we observed between microRNA and quantitative measurements of vascular function cannot simply be interpreted as causal. While longitudinal studies or

interventional trials could help establish a direct causal relationship, previous literature and our mediation analysis point towards a potential causal role of hub-microRNA miR-320 family on WMH burden through arterial compliance.

R1.5.2: *Comparison with Previous Literature:*
The manuscript does an excellent job of situating its findings within the broader context of existing literature. However, for discrepancies noted with other studies (e.g., miR-320e levels in relation to WMH load), a deeper exploration of potential reasons behind these differences could be enriched. Factors such as population demographics, microRNA isolation techniques, or measurement methodologies might offer explanatory insights that could guide future studies to reconcile these differences.

Answer: As mentioned in line 609, we believe that the discrepancy of our miR-320e with the findings of Gao and colleagues is due to different study designs and populations and different biomaterial used to isolate microRNAs.

R1.5.3: *Implications for Clinical Practice:*
While the manuscript outlines the potential implications of microRNA expression on vascular and cognitive health, expanding on how these findings could translate into clinical practice would significantly enhance the discussion. For instance, discussing the feasibility of leveraging microRNA profiles as biomarkers for early detection of vascular dysfunction or as therapeutic targets could provide a forward-looking perspective on the clinical relevance of this research.

Answer: We agree that it is important to highlight how our findings could translate into clinical practice. However, we have discussed this topic in the conclusions section, where we have summarized the key findings and their implication for future clinical practice.

R1.5.4: *Integrative Analysis and Biological Processes:*
The integrative analysis of microRNA and gene expression data is a highlight of the study, uncovering biological processes and pathways potentially regulated by the identified microRNAs. However, further discussion on the implications of these biological processes on vascular pathology and aging would be valuable. Additionally, considering indirect interactions or compensatory mechanisms within these pathways could offer a more comprehensive view of the vascular system's regulatory complexity.

Answer: The decision to consider only direct interactions (i.e., negative associations between candidate microRNA and predicted target gene) in the hub-microRNA – target genes analysis helps to focus on biologically relevant associations. MicroRNAs bind to target genes, leading to either degradation or inhibition of its translation into protein. These interactions are considered to have a more specific regulatory effect on the target gene. Therefore, by including only direct associations, we can identify more precise regulatory pathways and reduce noise derived from indirect associations.

R1.5.5: *Novelty and Contribution to the Field:*
The discussion underscores the novelty of the findings, particularly in identifying microRNAs not previously associated with vascular dysfunction. Emphasizing the contribution of this research to filling knowledge gaps, such as the role of newly identified

microRNAs in vascular health, would reinforce the study's significance.
Highlighting how this research expands the understanding of microRNA-mediated regulation of vascular functions could further articulate the novelty aspect.

Answer: We are limited in our ability to expand the discussion regarding novel microRNAs due to the lack of previous knowledge about them. Future studies, in both animal or cell models, are necessary to explore the distinct biological roles and mechanisms of these microRNAs in vascular health.

R1.5.6: *Future Research Directions:*
While the discussion briefly mentions future research avenues, elaborating on specific hypotheses or study designs prompted by these findings would be beneficial. For example, suggesting targeted functional studies to elucidate the mechanisms by which identified microRNAs influence vascular and cognitive health, or proposing the investigation of microRNA expression changes over time, could provide clear directions for subsequent research efforts.

Answer: We have briefly addressed this point in the limitations section, highlighting the need for longitudinal studies or interventional studies to elucidate the mechanisms by which candidate microRNAs influence vascular health.

R1.5.7: *Methodological Limitations:*
The acknowledgment of methodological limitations related to the candidate gene approach in the microRNA-gene expression analysis is important. Expanding on how future studies might overcome these limitations, perhaps by employing unbiased genome-wide approaches or integrating additional omics layers, would strengthen the discussion.

Answer: This limitation relates to including only direct interactions for functional analysis, while excluding indirect ones. However, it is ultimately the researchers' choice to decide whether to include only direct interactions or both direct and indirect interactions, depending mainly on their research questions.

R1.5.8: *Recommendations for Clinical and Research Applications:*
Finally, offering more concrete recommendations for clinicians and researchers based on the findings could enhance the manuscript's impact. For clinical applications, discussing potential screening or intervention strategies targeting identified microRNA pathways could be insightful. For research, suggesting methodological improvements or cross-disciplinary collaborations could help advance the field.

Answer: While we believe that our findings may be relevant for future clinical applications, we would prefer to give only a general overview on how these findings may translate into clinical settings, rather than providing specific recommendations.

In summary, the "Discussion" section effectively contextualizes the study's findings within the existing body of literature and identifies important implications for vascular and cognitive health. Addressing the above comments would further enhance the clarity, depth, and scientific rigor of the manuscript, ensuring it meets the high standards expected by prestigious journals.

More Comments on Discussions' Section

This discussion articulates the nexus between microRNA expression profiles and vascular health metrics, delineated through a population-based cohort study. The elucidation of associations involving the miR-320 family and other microRNA modules with vascular compliance and white matter hyperintensities (WMH) adds a pivotal chapter to the cardiovascular research narrative. Herein, I offer several profound, critical evaluations and recommendations, encapsulated in scientific verbiage:

1. *Data Validation*

The congruence of findings pertaining to the miR-320 family with prior literature corroborates the validity of the study's outcomes. Yet, the divergence observed in the case of miR-320e's relationship with WMH burden underscores the imperative for heterogenous data validation encompassing varied demographic and pathological cohorts. Enhanced validation could be sought through meta-analytical approaches or replication studies in cohorts with distinct ethnic and geographical backgrounds.

2. *Data Integration*

The integrative analysis of microRNA with gene expression data epitomizes a commendable methodological forte, facilitating the unveiling of potential regulatory pathways and target genes. Future endeavors could benefit from incorporating proteomic or metabolomic data, fostering a holistic multi-omics integration that could unravel the complex web of interactions underpinning vascular pathology.

3. *Strengths and Significance*

The population-based cohort study design, encompassing a wide age spectrum and employing a hypothesis-free approach, stands as a testament to the study's robustness. This methodology not only enriches the understanding of microRNA's role in vascular health but also broadens the horizon for identifying novel biomarkers for early detection and therapeutic targets.

4. *Novelty*

The identification of age-dependent associations of miR-6786-3p, miR-3605-3p, and miR-6747-3p with arterial compliance heralds a novel discovery, spotlighting microRNAs not previously implicated in vascular dysfunction. This novelty accentuates the importance of considering age as a differential factor in the molecular mechanisms of vascular aging and disease.

5. *Significance*

The elucidation of the miR-320 family's association with vascular health and cognitive performance delineates a significant stride towards understanding the molecular underpinnings of cerebrovascular and cognitive health. This discovery fosters a paradigm shift, suggesting microRNAs as not only biomarkers but also potential modulators of vascular and brain health.

6. *Drawbacks*

The cross-sectional design of the study introduces inherent limitations in establishing causality. Furthermore, the exclusive focus on negative associations in the integrative analysis of microRNA and gene expression might obfuscate potential indirect or positive regulatory interactions.

7. *Best Solution*

Longitudinal studies, employing causal inference methodologies such as Mendelian Randomization, could provide stronger evidence for the causal role of identified microRNAs

in vascular pathology. Additionally, expanding the analytical scope to include positive and indirect interactions in microRNA-gene expression analyses could unveil a more comprehensive regulatory landscape.

8. *Reproducibility*

While the study employs a rigorous methodological framework, the reproducibility of findings could be enhanced by making raw data and analytical pipelines accessible. Adoption of FAIR (Findable, Accessible, Interoperable, and Reusable) data principles would significantly contribute to this end.

9. *Potentially Further and Futuristic Research*

Future research could pivot towards exploring the therapeutic potential of modulating identified microRNAs, possibly through CRISPR/Cas9 technology or microRNA mimics/inhibitors. Investigating the interaction of lifestyle factors with microRNA expression could also yield insights into non-genetic modifiers of vascular health.

10. *Conclusions*

This investigation substantiates the pivotal role of microRNAs in vascular health, delineating their associations with arterial compliance and WMH burden, and posits their potential implications for cognitive health. These findings underscore microRNAs' multifaceted role in vascular pathology and cognition.

11. *Recommendations*

It is recommended that future studies embrace longitudinal designs, expand the scope of data integration, and focus on elucidating the causal pathways linking microRNA expression to vascular and cognitive health outcomes.

Moreover, translational research aimed at leveraging microRNAs as therapeutic targets warrants prioritization to translate these findings into clinical interventions.

R1.6: *Conclusion Section*
The conclusion succinctly encapsulates the key findings and implications of this comprehensive study on the role of circulating blood microRNAs in modulating vascular health and their extended influence on brain health. It commendably underscores the potential of microRNAs as biomarkers and therapeutic targets within the realm of cardiovascular diseases. Herein, I offer several critical, detailed, and constructive comments to enhance the scientific depth and clarity of this conclusion.

R1.6.1: *Articulation of Main Findings:*
The conclusion effectively summarizes the primary outcomes of the study, namely the association between specific microRNAs, notably the miR-320 family, and arterial stiffness, as well as the novel link to white matter hyperintensities (WMH) burden. However, it would be beneficial to explicitly mention the directness and strength of these associations, as well as the statistical confidence surrounding these findings, to provide a clearer picture of their robustness and reliability.

Answer: While we agree that reporting the direction and the strength of the associations is important, we have chosen to provide this information in the results section and to elaborate on the significance of the findings in the discussion section.
We would like to keep the conclusion as a synthesis of our study, summarizing the key findings.

R1.6.2: *Mechanistic Insights:*
While the conclusion alludes to the identification of biological processes involving target genes, a more detailed exposition of how these processes contribute to vascular dysfunction and the potential mechanistic pathways would enrich the reader's understanding. Highlighting specific pathways or target genes of interest could emphasize the novelty and depth of these findings, underpinning the molecular mechanisms that were elucidated.

Answer: Based on this comment, we have slightly adapted the sentence regarding biological processes, by including the most relevant pathways for vascular function:

Line 712: Additionally, we identified the biological processes in which the target genes are involved, such as blood vessel development and angiogenesis, thereby enhancing the understanding of the molecular mechanisms underlying vascular (dys)function.

R1.6.3: *Implications for Brain Health:*
The mention of the association between miR-320 and WMH burden is pivotal, yet the conclusion could further benefit from a discussion on the broader implications of this relationship for brain health. Elaborating on how these findings integrate with current understanding of cerebrovascular pathophysiology and cognitive decline could illuminate potential interdisciplinary impacts of the study.

Answer: Similar to comment **R1.6.1**, in order to keep the conclusion as a synthesis of our study, we have elaborated on the possible implications of the association between miR-320 and WMH burden in the discussion section (line 598).

R1.6.4: *MicroRNAs as Therapeutic Targets:*
The conclusion posits microRNAs as potential targets for novel preventive strategies against cardiovascular diseases, a statement of significant clinical relevance. Expanding on the feasibility, challenges, and current status of microRNA-based therapies in cardiovascular medicine could offer valuable context and highlight the translational potential of these findings.

Answer: Similar to comment **R1.6.1**, in the conclusion section, we would like to mainly highlight the key findings and provide a general overview of how these findings may translate into clinical settings. Expanding on the feasibility, challenges and current status of microRNA-based therapies in cardiovascular medicine requires an extensive literature review, which is beyond the scope of the conclusion section.

R1.6.5: *Future Research Directions:*
While the conclusion aptly summarizes the study's contributions, incorporating specific recommendations for future research endeavors could further enhance its utility. Suggestions could include the need for longitudinal studies to confirm causality, the exploration of microRNA-based interventions, and the investigation of the combined effect of multiple microRNAs on vascular and brain health.

Answer: We agree that incorporating specific recommendations for future studies could enhance its utility. However, we have chosen to include these suggestions within the limitations section. In fact, we have provided suggestions on how future research may address the limitations of the study, such as utilizing longitudinal or interventional trials to

establish the direct causal relationship between microRNAs and vascular health (line 681).

R1.6.6: *Clinical and Public Health Relevance:*
A brief discussion on the potential impact of these findings on clinical practice and public health strategies would provide a compelling closure to the conclusion. Mentioning how these insights could inform risk stratification, personalized medicine, or the development of public health interventions could underscore the real-world applicability of the study's outcomes.

Answer: To address this comment, we have slightly adapted the sentence regarding using candidate microRNAs for the prevention of cardiovascular diseases:

Line 714: Overall, our findings highlight the crucial role of microRNAs as key regulators of vascular health, implicating them as potential targets for the development of novel preventive strategies against cardiovascular diseases in clinical settings.

In sum, the conclusion effectively encapsulates the essence and implications of the study's findings, positing microRNAs as pivotal elements in the nexus between vascular and brain health. Addressing the above comments could enhance the conclusion's depth, providing a clearer roadmap for future research and potential clinical translation of these significant findings.

Answer to Reviewer 2

R2.1: *61: Four modules, represented by hub-microRNAs miR-320 family, miR-378 family, miR-3605-3p, miR-6747-3p, miR-6786-3p, and miR-330-5p, were associated with total arterial compliance index.*
This sentence is confusing. It says four modules were represented by hub miRNAs but then lists six miRNAs. I would expect one (central) hub for each module.

Answer: We understand that this sentence might confuse the reader. Therefore, we have slightly modified the abstract.
We have added a sentence to clarify that one module may have more than one microRNA:

Line 56: Through linear regression models, we investigated the association between each module's expression and quantitative markers of vascular health, including pulse wave velocity, total arterial compliance index, cardiac index, stroke index, systemic vascular resistance index, reactive skin hyperemia and white matter hyperintensity burden. For each module associated with at least one trait, one or more hub-microRNAs driving the association were defined. Hub-microRNAs were further characterized through mapping to putative target genes followed by gene ontology pathway analysis.

In the methods section, the reader will find the definition of the hub-microRNAs, which based on your next suggestions, we have separated into one separate subsection:

Line 337: *Identification of hub-microRNAs for each module*
For each key module, defined as a module significantly associated with at least one of the investigated traits, hub-microRNAs were identified. We defined hub-microRNAs as microRNAs that a) had module membership greater than 0.8, and b) were significantly associated with the selected trait (p-value < 0.05). Therefore, hub-microRNAs served as module representatives and were the main drivers of the association between the module and the candidate trait.

R2.2: *116: This panel included also white matter hyperintensities (WMHs), an imaging marker of cerebral small vessel disease, enabling exploration of the potential link among microRNAs, vascular function and brain health.*
The paper uses WMH abbreviation and results a lot, but it was never actually explained what exactly are white matter hyperintensities, what is their burden, and why are they relevant. The description in the Methods section is too technical to substitute for the proper explanation in the Introduction or Results section. Please bear in mind that the paper may be read by scientists from the field of cardiovascular diseases who are not familiar with specific neurobiological methodologies.

Answer: We agree that for someone who is not familiar with neurology, neuroscience or the cardiovascular system, it may be difficult to understand what WMHs are and why they are relevant to cardiovascular health.
Therefore, we have added in the background section a short paragraph which puts WMH into context:

Line 123: This panel included also white matter hyperintensities (WMHs), the most common magnetic resonance imaging (MRI) marker of cerebral small vessel disease [20]. The prevalence and extent of WMHs strongly increase with age, and they are associated with an increased risk of cognitive decline, dementia and stroke [21]. Including this measurement in the vascular panel allowed for the exploration of potential links among microRNAs, vascular function and brain health.

R2.3: *353: higher eigen-microRNA vectors of the black and midnightblue modules were associated with a worse total arterial compliance index (□□ estimate = -0.039, **FDR = 0.063** and □□estimate = -0.054, FDR = 0.007, respectively). Consistent with the above results, we found a larger eigen-microRNA vector of the black module to be associated with an increase in WHM burden (□□ estimate = 0.047, **FDR = 0.053**).*
What is the reason to present findings that have not reached the threshold of statistical significance? If it is absolutely necessary, valid justification should be provided, otherwise these results should be removed.

Answer: We acknowledge that our wording in the manuscript was not totally clear. Therefore, we take the chance to explain our reason to present findings which are FDR>0.05.

The lower a p-value, the less likely it is that observed associations were due to chance without there being a true relationship. Although the threshold of statistical significance is commonly set at 0.05, this is an arbitrary threshold. Findings with a p-value closer to this threshold are less likely to be due to chance than findings with a much larger p-value. For this

reason, we also reported, as borderline significant, findings that were very close to the FDR<0.05 but did not pass the threshold.

In line with this, we have modified the method section about the statistical significance threshold, accordingly:

Line 332: Multiple testing correction was applied using the Benjamini-Hochberg false discovery rate (FDR) method [39]. We reported results both at the FDR < 0.1 and FDR < 0.05 thresholds. This was done to avoid rejecting as statistically non-significant results that were very close to the common FDR < 0.05 threshold, thus reducing the possibility of false negatives (Type II error).

In the limitation paragraph, we have included a sentence explaining that the results which are borderline to the statistical significance threshold at FDR<0.05 might be attributable to the limited statistical power due to the sample size. We are reporting the sentence below:

Line 686: Third, although our study is one of the biggest studies to date that investigates the relationship between microRNAs and vascular health, it might still have limited power to detect smaller effect sizes, due to insufficient sample size. Therefore, future studies are necessary to validate our findings, especially the ones which did not reach the conventional threshold of statistical significance at FDR<0.05.

R2.4: *373: In the cyan module, miR-330-5p had the highest module membership value, and because it showed a borderline significant association with total arterial compliance index (module membership = 0.816, p-value = 0.056), we decided to include it as a hub-microRNA The same comment as above.*

Answer: We agree that this aspect was not clearly explained in the manuscript. Therefore, we have now revised the corresponding section accordingly. Specifically, we have clearly stated that for the *cyan* module, there were no microRNAs that met our criteria to be defined as hub-microRNAs. We decided to consider miR-330-5p as a hub-microRNA in order to further explore this module through downstream analysis, given its significant association with total arterial compliance index. Specifically, miR-330-5p was selected because it had the highest module membership value and it showed a borderline significant association with total arterial compliance index.

We are reporting the edited section below:

Line 472: Within the cyan module, none of the microRNAs fulfilled the criteria to be defined as a hub-microRNA. However, to be able to further explore this module, which was significantly associated with total arterial compliance index, we considered miR-330-5p as the hub-microRNA. This selection was based on its proximity to satisfying both hub-microRNA criteria (i.e., module membership and association with the outcome). Specifically, miR-330-5p had the highest module membership value (0.816) and it showed a borderline significant association with total arterial compliance index (p-value = 0.056).

R2.5: *438: We identified associations with total arterial compliance for four microRNA modules, represented by the miR-320 family, miR-378 family, miR-6786-3p, miR-6747-*

3p, miR-3605-3p and miR-330-5p.
Again, the same comment as the first one. This sentence is confusing. It says four modules were represented by hub miRNAs but then lists six miRNAs. I would expect one (central) hub for each module. So, either four miRNAs should be listed or this whole relationship between modules and hubs clearly explained.

Answer: Based on previous suggestions, comments and the current feedback, we have carefully edited some sections in the manuscript to make the relationship between modules and hub-microRNAs clearer and easier for the reader to follow. We report the changes we made below.

We have created a subsection dedicated to hub-microRNAs in both the methods and results sections, which we hope will facilitate the reader to grasp the idea of hub-microRNAs, which is crucial for understanding the manuscript. The subsection is called "Identification of hub-microRNAs for each module".

Moreover, the methods subsection about hub-microRNAs was adapted in the following manner:

Line 338: For each key module, defined as a module significantly associated with at least one of the investigated traits, hub-microRNAs were identified. We defined hub-microRNAs as microRNAs that a) had module membership greater than 0.8, and b) were significantly associated with the selected trait (p-value < 0.05). Therefore, hub-microRNAs served as module representatives and were the main drivers of the association between the module and the candidate trait.

At the beginning of the "Identification of hub-microRNAs for each module" subsection in the results, we included the following sentence to emphasize that a single module can have several hub-microRNAs:

Line 462: Next, we detected the hub-microRNAs for each key module, defined as any microRNAs that had high module membership values and were significantly associated with the corresponding trait, as depicted in Fig. 3 (Table 2).

R2.6: *465: Importantly, another study carried out by our group has provided suggestive evidence that higher miR-320 expression levels are also related to worse cognitive performance (Melas K. et al, Circulating microRNAs are related to cognitive performance in the general population, Unpublished),*
Do the journal rules allow citing unpublished research?

Answer: We have now uploaded the manuscript into MedRxiv and cited it as follows:
Melas K, Talevi V, Etteldorf R, Estrada S, Krüger DM, Pena T, et al. Circulating microRNAs are related to cognitive domains in the general population. medRxiv. 2024:2024.05.07.24306994 [#55]

R2.7: *First, it was based on cross-sectional observational data, and the relations we observed between microRNA and quantitative measurements of vascular function cannot*

simply be interpreted as causal.
The authors may try using software for causal inference, such as TETRAD (https://www.cmu.edu/dietrich/philosophy/tetrad/). However, I realise that mastering this program may be outside the timeframe for this review. Rather, the authors may try to use it in their future research.

Answer: Thank you for your suggestion. We will consider this website for future analyses.

R2.8: *545: any -> some*

Answer: We have changed it. Thank you for pointing that out.

R2.9: *Fig2: both FDR<0.1 and FDR<0.05 are marked with one star. This is very confusing. As I suggested above, FDR<0.1 should not be shown or it has to be justified and shown with a clearly distinct symbol.*

Answer: We agree that the symbols and the sizes we used were not entirely suitable for clearly distinguishing between FDR<0.05 and FDR<0.1.
Therefore, we have modified Fig2 by replacing the big dot representing FDR<0.1 with a small dot, allowing it to be immediately differentiated from the star indicating FDR<0.05.

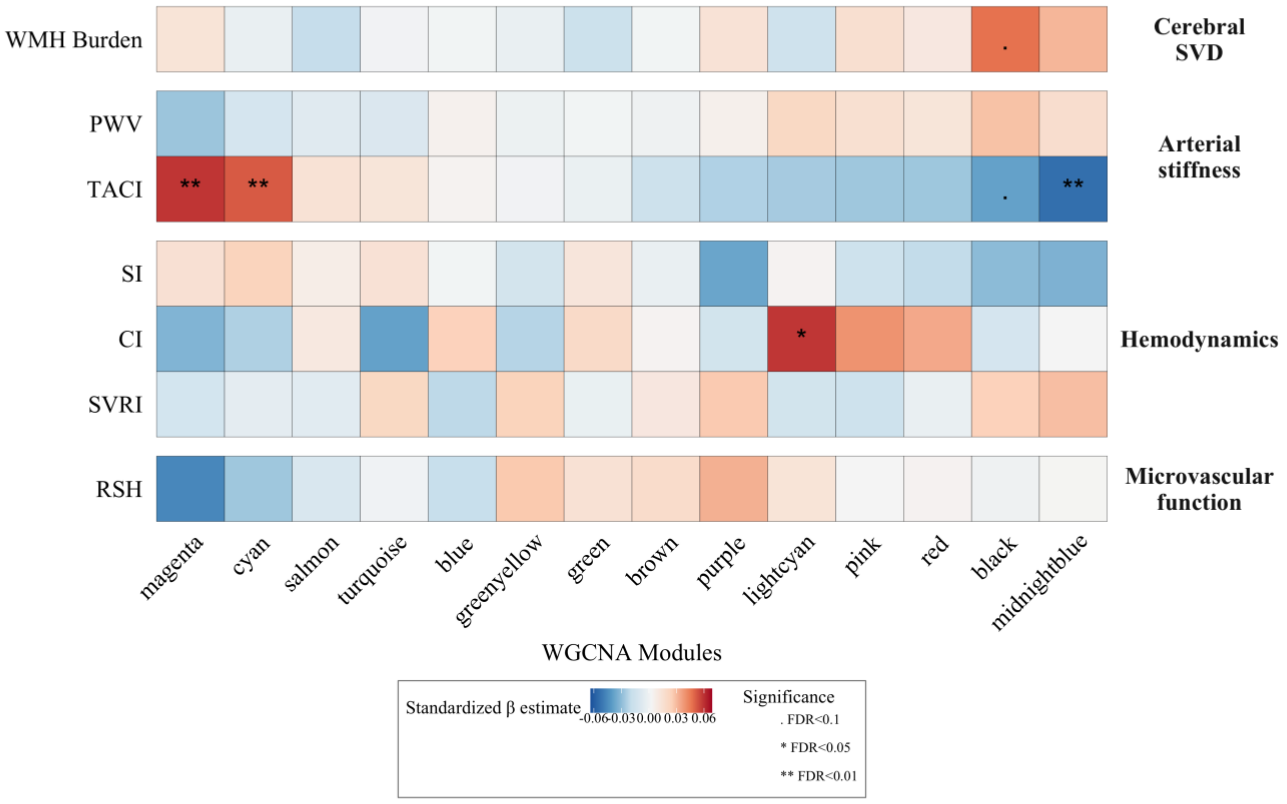

Additionally, we have adjusted supplementary figure 4 (**Fig. S4**) to match the legend symbols used in **Fig. 2**.

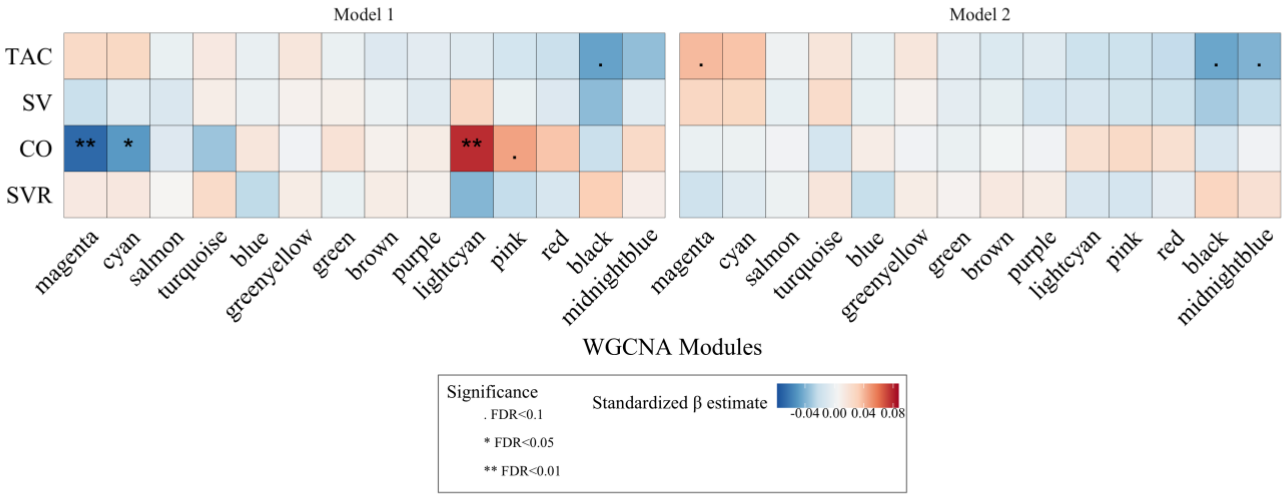

R2.10: *Table2: miR-330-5p association with Total arterial compliance index is not significant and this row should be removed*

Answer: Based on your previous comments and suggestions, we have explained in the manuscript that although miR-330-5p did not completely fulfill the requirements to be defined as a hub-microRNA, it was selected as the hub-microRNA for the *cyan* module. This allows for further exploration of the *cyan* module through downstream analyses.
Therefore, we would like to keep miR-330-5p in the table as hub-microRNA.

R2.11: *Table3: I would suggest sorting the miRNAs by the number of Confirmed target genes rather than Percentage of confirmed genes.*

Answer: We have followed your suggestion and adapted the table 3 accordingly.

**Hub-microRNAs**	**Predicted target genes[a]**	**Confirmed target genes[b]**	**Percentage of confirmed genes**
miR-320b	1621	502	31.0
miR-320c	1572	481	30.6
miR-320d	1540	477	31.0
miR-330-3p	1330	458	34.4
miR-6747-3p	1351	414	30.6
miR-320a-3p	1069	358	33.4
miR-192-5p	1311	260	19.8
miR-378a-3p	619	155	25.0
miR-378c	494	118	23.9
miR-378i	488	115	23.6

miR-378f	489	110	22.5
miR-6786-3p	52	13	25.0
miR-3605-3p	58	11	19.0

R2: *This is an important study presenting valuable data on a large human cohort, however the presentation of the results is not optimal. There is no clear structure in the Results section and in the Discussion, making it very hard to follow. A reader will lose track of multiple miRNA names which are just numbers not familiar to a general reader and hard to remember, unlike gene names. Perhaps, it would make sense to discuss each miRNA in its own separate subsection, both in the Results and in the Discussion, or to find another way to present results in a clear and well-structured manner.*

It seems that including results with FDR below the widely accepted 0.05 significance level allowed the authors to include the black module and WMH (see Fig2). Given that WMH were not properly explained, I suggest that removing both the black module and WMH results will streamline the paper and make it more rigorous. However, if the authors feel that the black module and WMH are important to report on, the non-standard FDR threshold should be clearly justified and highlighted in the Results section, and WMH relevance should be explained in the Introduction.

Answer: Thank you for your suggestions and comments. In summary, by following your advices, we have adapted the manuscript in the following way.
We have included a section regarding WMH and its relation to vascular function to put WMH burden into context. In addition, we have carefully explained in the methods section the rationale behind of including results which are borderline with the conventional threshold of statistical significance (0.05). To better differentiate results which reached the threshold of statistical significance from the ones that were borderline to the threshold of 0.05, we adapted Fig. 2 and Supplementary Fig. S4, as suggested. Moreover, we have edited methods and results sections in the manuscript, by dedicating a specific subsection for clearly explaining what is a hub-microRNA, the criteria that define a hub-microRNA and its relation with the corresponding module. Finally, in the result section, we have included short explanatory sentences which introduce each subsection and put results into context. We hope this will make the manuscript's structure and our findings easier to follow.
Thank you for your input and suggestions on how to make the manuscript clearer.

Answer to Reviewer 3

R3: *The study "Peripheral whole blood microRNA expression in relation to vascular function: a population-based study" presents valuable findings about, however if the following comments are answered and incorporated into manuscript, it would enhance the impact of the research.*

R3.1: *Could you elaborate on the selection criteria for participants in the Rhineland Study, especially regarding any pre-existing cardiovascular conditions?*

Answer: We agree that we should elaborate further the criteria for participants in the Rhineland Study and specify pre-existing cardiovascular conditions.
Therefore, we edited the manuscript in the following manner:
1) In the methods section, we have better specified that there are no specific selection criteria for the inclusion of participants in the Rhineland Study, a community based prospective cohort study. The participants represent mainly the general population. The only requirements are that the participants are aged 30 years and above and have sufficient German language skills to provide informed consent.
Following the edited text in the manuscript:

Line 142: We used data from the Rhineland Study, an ongoing population-based prospective cohort study in Bonn, Germany. All inhabitants of two geographically-defined areas of Bonn are invited to participate in the Rhineland Study. There are no specific selection criteria. The only requirements are that participants are aged 30 years and above, and have sufficient command of the German language to provide informed consent.

2) In the results section, we have included the number of participants with pre-existing cardiovascular conditions in the following way:

Line 413: The characteristics of the study population are reported in Table 1. The analysis included 2606 participants with available microRNA expression data and cardiovascular measurements. The mean (± standard deviation) age of the participants was 53.93 ± 13.95, including 1194 men (45.8%). Among the participants included in the study, the following cardiovascular conditions were self-reported: stroke (n = 50), heart failure (n = 65), coronary artery disease (n = 145), hypertension (n = 1076), arrhythmia (n = 336), heart valve disease (n = 107), myocardial infarction (n = 54) and peripheral arterial disease (n = 40).

R3.2: *How did you account for potential confounding factors, such as lifestyle or genetic predispositions, in your analysis of microRNA associations with vascular health?*

We believe that smoking, as a lifestyle factor, and genetic predispositions are relevant both for microRNAs and vascular function. However, we don't think these two factors can simply be treated as confounders. Although smoking and genetic predispositions might affect both microRNAs and vascular function, it is very likely that the microRNAs are in the causal pathways of the relation between smoking/genetic predispositions and vascular function. Unravelling the exact nature of the relations between smoking, genetic predisposition, microRNAs and vascular function, and how they interact with each other, is outside the scope of the current paper, as it would require extensive additional analyses and, possibly, different research settings (e.g. experimental studies).

Nevertheless, we now conducted two different sensitivity analyses.
In the first analysis, we have further adjusted for smoking status, which is one of the main lifestyle risk factors for cardiovascular disease.

After the adjustment for smoking status, we observed that the associations are comparable with the main model, which was only adjusted for age and sex, suggesting that the relationships between microRNA modules and vascular traits are independent of smoking.

We have reported below a direct comparison of the results obtained from the main model, used in the manuscript and the one adjusted for smoking.

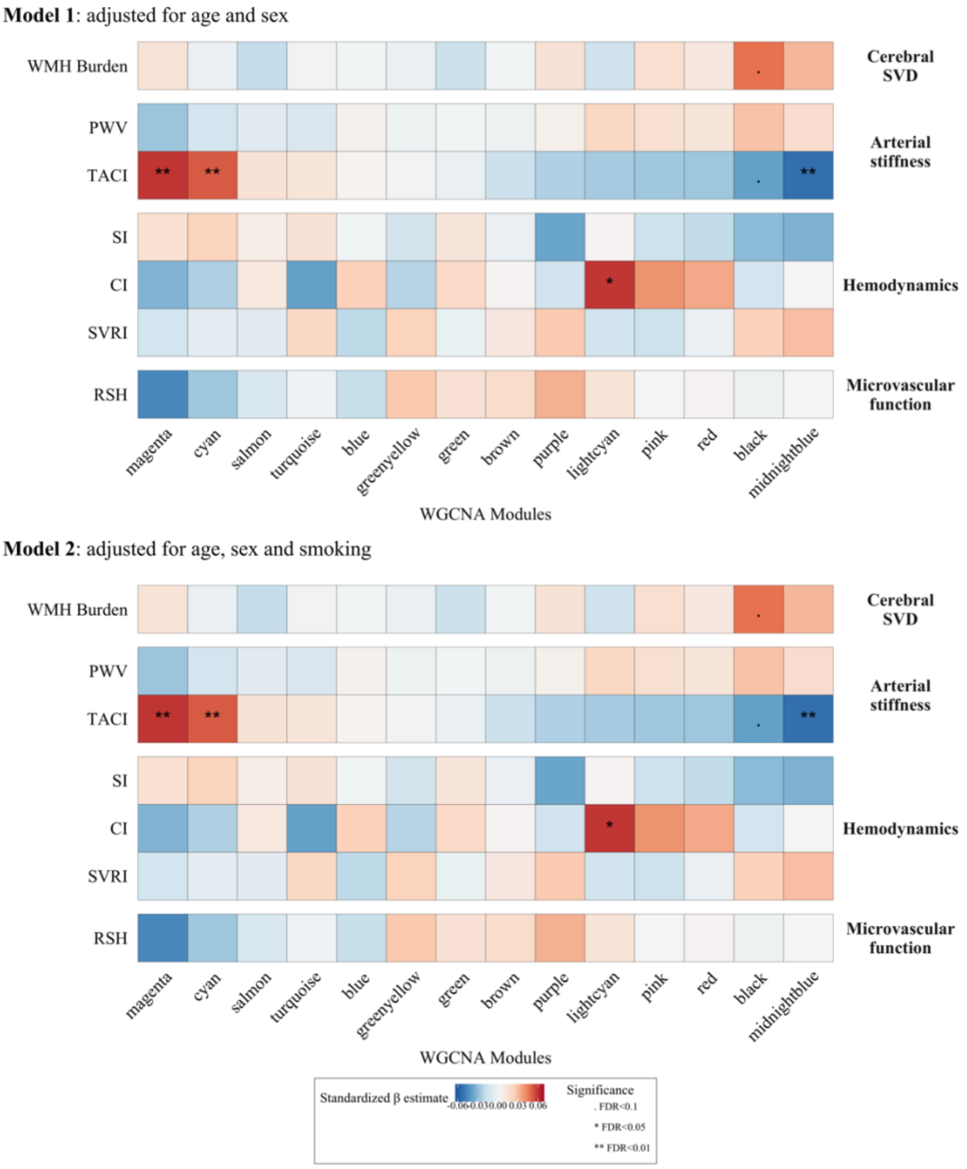

Model 1								
**Trait**	**Module**	**Estimate**	**p.value**	**Lower95**	**Upper95**	**Fstatistic**	**t_value**	**p.adjusted**
TACI	Cyan	0.05157	0.00147	0.0198	0.08333	410.57266	3.18319	0.00688
TACI	Magenta	0.05868	3e-04	0.02689	0.09047	412.02628	3.61975	0.00421
TACI	Black	-0.0385	0.01805	-0.07041	-0.00659	408.35343	-2.36615	0.06316
TACI	Midnightblue	-0.05359	0.00114	-0.08585	-0.02134	410.80925	-3.25823	0.00688
CI	Lightcyan	0.05865	0.00144	0.0226	0.09469	125.55667	3.19064	0.02011
WMH burden	Black	0.04705	0.00377	0.01524	0.07886	579.28769	2.90057	0.05271
**Model 2**								
**Trait**	**Module**	**Estimate**	**p.value**	**Lower95**	**Upper95**	**Fstatistic**	**t_value**	**p.adjusted**
TACI	Cyan	0.05161	0.00145	0.01987	0.08336	309.21878	3.18782	0.00677
TACI	Magenta	0.06078	0.00018	0.02895	0.0926	310.63873	3.74495	0.00258
TACI	Black	-0.03738	0.02172	-0.06929	-0.00547	307.422	-2.29668	0.07601
TACI	Midnightblue	-0.0549	0.00086	-0.08716	-0.02265	309.57769	-3.33744	0.006
CI	Lightcyan	0.05778	0.00173	0.02166	0.0939	94.28933	3.13689	0.02417
WMH burden	Black	0.0463	0.0044	0.01445	0.07814	434.69353	2.85115	0.06161

In the second analysis, we have adjusted the association between microRNAs and vascular health for genetic predispositions. Specifically, we used polygenic risk scores (PRS) generated by previous studies for the following cardiovascular diseases:

1) Angina (Jung H et al. Commun Biol, 2024)

2) Acute myocardial infarction (Jung H et al. Commun Biol, 2024)

3) Pulmonary embolism (Jung H et al. Commun Biol, 2024)

4) Heart failure (Jung H et al. Commun Biol, 2024)

5) Cerebral infarction (Truong B et al. Cell Genom, 2024)

6) Atherosclerosis (Privé F et al. Am J Hum Genet, 2022)

7) Hypertension (Jung H et al. Commun Biol, 2024)

8) Peripheral vascular disease (Privé F et al. Am J Hum Genet, 2022)

9) Coronary artery disease (Jung H et al. Commun Biol, 2024)

The following model was used:

Vascular trait$_y$ ~ intercept + microRNA-module$_i$ + age + sex + PRS_Angina + PRS_AcuteMyocardialInfarction + PRS_PulmonaryEmbolism + PRS_HeartFailure + PRS_CerebralInfarction + PRS_ Atherosclerosis + PRS_ Hypertension + PRS_PeripheralVascularDisease + PRS_CoronaryArteryDisease + 10_PrincipalComponents

After adjusting for PRSs, we observed that the associations between magenta, cyan and midnightblue modules and the total arterial compliance index remained significant, and their directions were consistent with those in model 1. The associations of the black module with WMH burden and with total arterial compliance index, as well as the association between the

lightcyan module and cardiac index, no longer reached statistical significance, although the estimates only slightly changed from those observed in model 1. We think that the lower number of significant associations is primarily due to the inclusion of 19 additional covariates in the model, which consequently decreases the statistical power. This may have especially impacted the power for the analyses on WMH, where we had a smaller sample size.

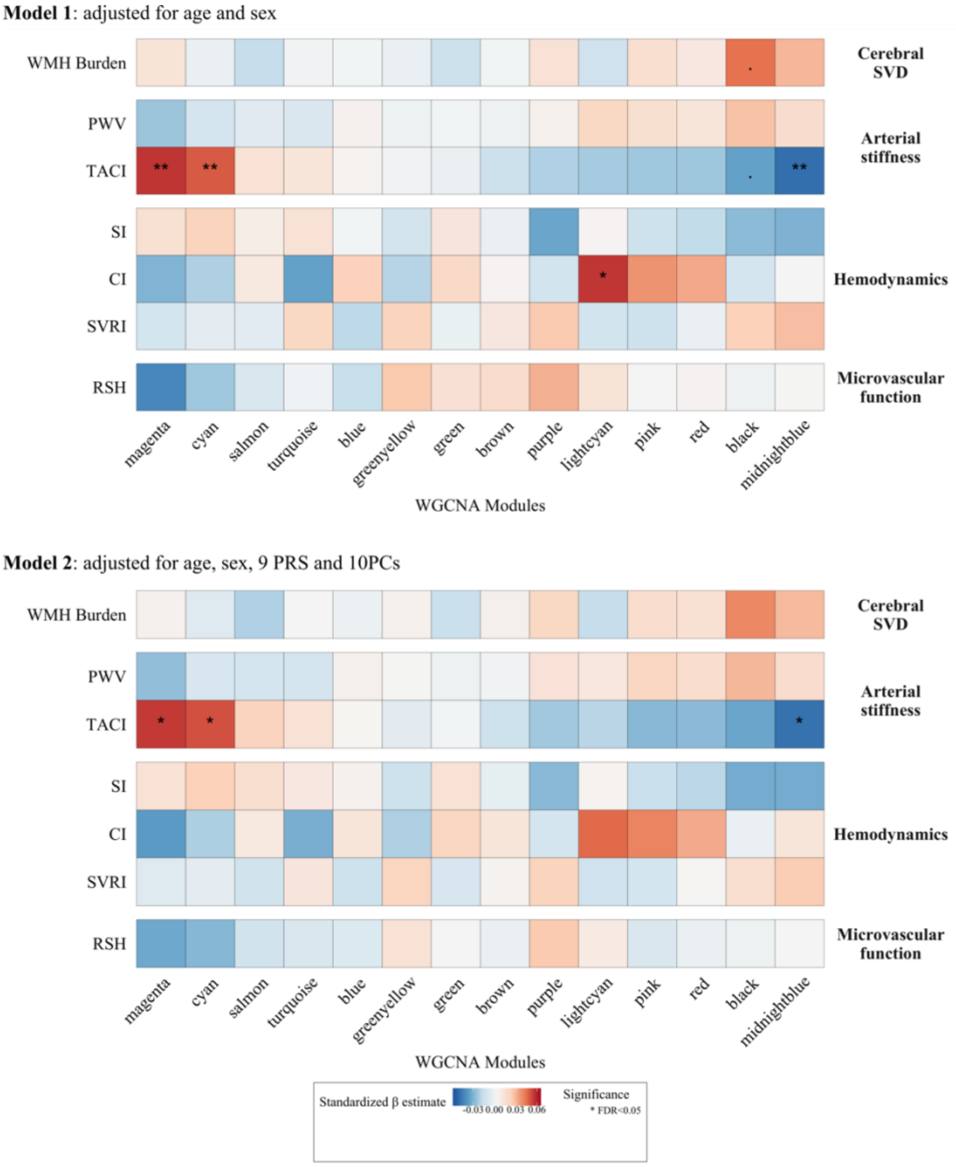

Model 1								
**Trait**	**Module**	**Estimate**	**p.value**	**Lower95**	**Upper95**	**Fstatistic**	**t_value**	**p.adjusted**
TACI	Cyan	0.05157	0.00147	0.0198	0.08333	410.57266	3.18319	0.00688
TACI	Magenta	0.05868	3e-04	0.02689	0.09047	412.02628	3.61975	0.00421
TACI	Black	-0.0385	0.01805	-0.07041	-0.00659	408.35343	-2.36615	0.06316
TACI	Midnightblue	-0.05359	0.00114	-0.08585	-0.02134	410.80925	-3.25823	0.00688
CI	Lightcyan	0.05865	0.00144	0.0226	0.09469	125.55667	3.19064	0.02011
WMH burden	Black	0.04705	0.00377	0.01524	0.07886	579.28769	2.90057	0.05271

Model 2								
**Trait**	**Module**	**Estimate**	**p.value**	**Lower95**	**Upper95**	**Fstatistic**	**t_value**	**p.adjusted**
TACI	Cyan	0.05106	0.00306	0.01729	0.08483	53.31854	2.96529	0.02139
TACI	Magenta	0.05538	0.0013	0.02166	0.08909	53.42759	3.22105	0.01814
TACI	Black	-0.03396	0.04707	-0.06748	-0.00044	52.98456	-1.98679	0.16473
TACI	Midnightblue	-0.04877	0.0048	-0.08265	-0.01489	53.26182	-2.82313	0.02239
CI	Lightcyan	0.04642	0.01945	0.00749	0.08535	15.87455	2.33846	0.27231
WMH burden	Black	0.04016	0.01889	0.00664	0.07368	71.78839	2.34996	0.26441

Based on the explanation given above, that we don't think that smoking and genetic predispositions can be treated as confounders, we would prefer not to include all the sensitivity analyses for publication in the manuscript.

R3.3: *Can you clarify the role of miR-320 in cerebrovascular damage mediation, particularly how it influences arterial compliance?*

Answer: To clarify the role of miR-320 family in cerebrovascular damage mediation through arterial compliance, we added the following statement in the manuscript, which summarizes our findings about miR-320:

Line 594: Notably, our results suggest that the relation of the miR-320 family with vascular function has even implications for brain health, as higher expression of this molecule was also related to increased WMH burden, a biomarker of cerebral small vessel disease. This notion was strengthened by our mediation analysis, which showed that total arterial compliance partially mediates the effect of miR-320 on WMH burden. Taken together, these findings suggest that an increase of miR-320 expression levels in the blood leads to a decrease in total arterial compliance, affecting the functioning of endothelial and vascular smooth muscle cells, as shown in previous studies [49-51]. The increased arterial stiffness can lead to impaired vascular function and blood-brain barrier disruption, ultimately contributing to the development of WMHs.

R3.4: *Were there any significant differences in microRNA expression or vascular health markers between age groups within your study cohort?*

Answer: To answer this question, we have included scatter plots (**Fig. S3**) in the manuscript. These plots show the relationship between age and vascular function traits, as well as microRNA-related modules.

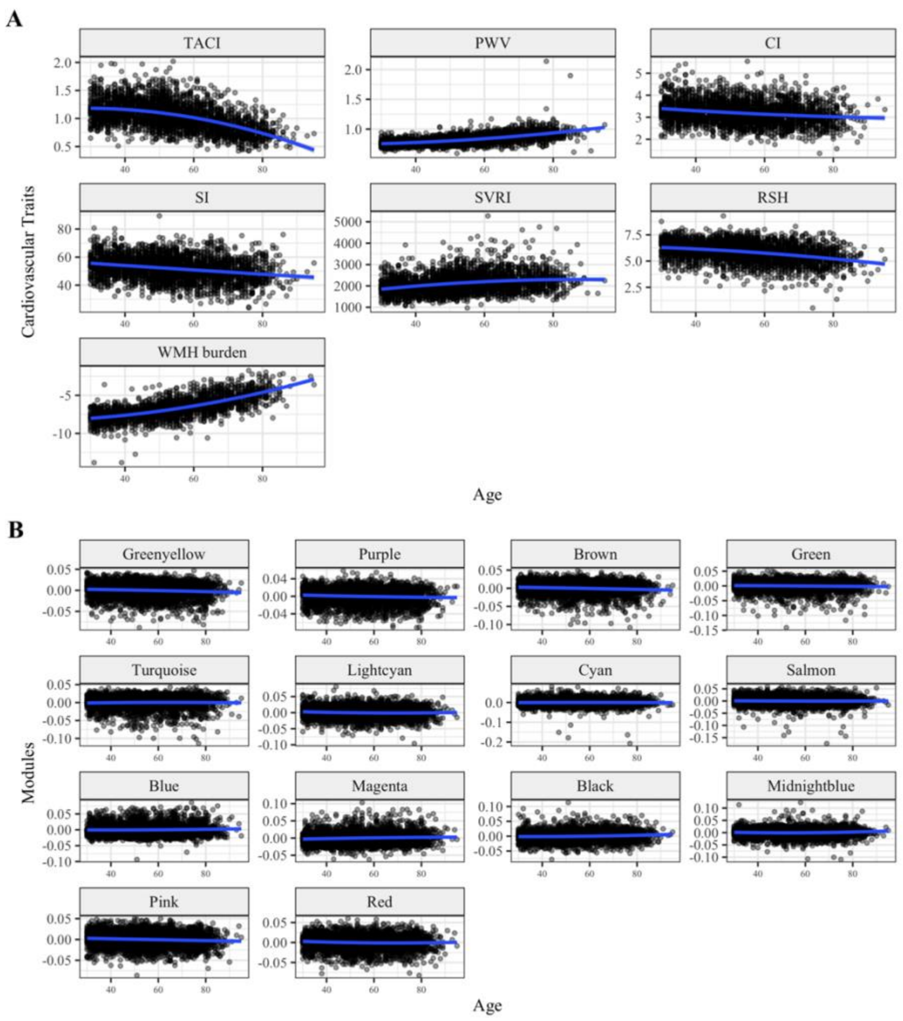

We have adapted the methods and the results sections accordingly.
Methods section:

Line 320: As a first exploratory analysis, we evaluated the relationship between age and cardiovascular measurements, as well as microRNA modules.

Results section:

Line 435: The exploratory analysis showed the expected relationships between age and vascular measurements: total arterial compliance index, cardiac index, stroke index and reactive skin hyperemia decreased with age, while pulse wave velocity, systemic vascular resistance index and WMH burden increased. In contrast, the eigen-microRNA vectors were relatively stable across age (**Additional file 1: Fig. S3**).

R3.5: *Regarding the functional analysis, how did you determine the specificity of the identified pathways to the vascular function, and could these pathways also be relevant to other biological processes or diseases??*

Answer: Thank you for the question. We first selected all the pathways that reached the significant level at p.adjusted<0.05. Then we pointed out pathways potentially involved

in vascular function, based on the literature. Based on your question, we have adapted the corresponding section in the manuscript:

Line 387: The p-values of the terms within each group were combined using the Fisher method and multiple testing correction was applied using the Benjamini-Hochberg false discovery rate (FDR) method [39]. Only terms that reached the significant level at FDR < 0.05 were reported.

R3.6: *Cite the following related articles in related work or introduction to give the strong literature base.*

- ➔ *A systematic review of computational approaches to understand cancer biology for informed drug repurposing (https://doi.org/10.1016/j.jbi.2023.104373)*
- ➔ *Drug Repurposing for viral cancers: A paradigm of machine learning, deep learning, and Virtual screening- based approaches (https://doi.org/10.1002/jmv.28693)*
- ➔ *Drug repurposing in psoriasis, performed by reversal of disease-associated gene expression profiles (DOI: 10.1016/j.csbj.2022.10.046)*
- ➔ *A comprehensive review of artificial intelligence and network based approaches to drug repurposing in Covid-19 (DOI: 10.1016/j.biopha.2022.113350)*
- ➔ *SperoPredictor: An Integrated Machine Learning and Molecular Docking-Based Drug Repurposing Framework With Use Case of COVID-19 (https://doi.org/10.3389/fpubh.2022.902123)?*
- ☐ *Network-based drug repurposing for HPV- associated cervical Cancer (DOI: 10.1016/j.csbj.2023.10.038)* ☐ *Network-based drug repurposing identifies small molecule drugs as immune checkpoint inhibitors for endometrial cancer (https://link.springer.com/article/10.1007/s11030-023-10784-7)*
- ☐ *Artificial Intelligence Assisted Repurposing of Lubiprostone Alleviates Tubulointerstitial Fibrosis (https://www.sciencedirect.com/science/article/pii/S1931524423001287?via%3Dihub)*
- ☐ *A comprehensive review of key factors affecting the efficacy of antibody drug conjugate (https://www.sciencedirect.com/science/article/pii/S0753332223001968?via%3Dihub)*
- ☐ *Microphysiological System with Continuous Analysis of Albumin for Hepatotoxicity Modeling and Drug Screening (https://www.sciencedirect.com/science/article/pii/S1226086X21001581?via%3Dihub)*

Answer: We have carefully examined the suggested papers. These papers focus on multi-omics-based approaches, network-based approaches, pathway-based approaches, machine learning and deep learning. However, the main goal of these papers is using these methods for drug repurposing, specifically in cancer, COVID-19 and psoriasis. Although they present interesting methods, we could not see how these papers would be relevant to our manuscript and its goals. We are happy to receive suggestions on which specific sections of our manuscript would be improved by referencing these papers, as well as on any other papers that may strengthen our manuscript.

Answer to Reviewer 4

R4: *This is a well-written paper containing interesting results which merit publication. The abstract summarizes the full text in a relatively concise and clear manner; the authors provide detailed background information; the tables and figures in the text are clear and unambiguous; the potential value of the study is clear; the methodology of the study is clear and the structure of the article is well laid out; the algorithms, methodologies, experiments, and conclusions of the study are reliable, comprehensive, and reasonable; the authors indicate the limitations of the study.*

Answer: Thank you for your review.

Answer to Reviewer 5

R5: The methodology section has several shortcomings that need to be addressed:

R5.1: *Due to the unavailability of data to the public, reproducing the study would be challenging.*

Answer: The data are not publicly available due to data protection regulations. However, the data can be conditionally provided to applying scientists in accordance with Rhineland Study's Data Use and Access Policy.

R5.2: *Conducting a power analysis is necessary to ensure the population size is sufficient for the study.*

Answer: Observational studies usually do not require a priori power size calculation. Sample sizes in observation studies are typically based on the largest complete dataset that can be obtained. However, we have conducted a post-hoc power analysis using the pwr package in R.
Having a sample size of N = 2606, a significance level set at 0.05, a power parameter equal to 0.8 and numerator degrees of freedom equal to 2, it is sufficient to detect associations with very small effect sizes ($f^2 = 0.0037$).
We would prefer not to include this analysis in the manuscript, as it is not a type of analysis commonly reported in observational studies.

R5.3: *The exclusion criteria lack clarity, and it's important to explain why the sample size was reduced from 3000 to 2606 participants.*

Answer: Following your comment, we have included in the method section the exclusion criteria, which we report below:

Line 160: In this study, we used baseline data of the first 3000 consecutive participants of the Rhineland Study. MicroRNA expression data were unavailable for 33 individuals due to

technical issues, with an additional 38 participants being excluded during quality control procedures. Moreover, 323 participants were excluded because of missing cardiovascular data due to contraindication/exclusion criteria (n = 246), participant refusal (n = 8), technical problems (n = 12), exclusion during quality assurance (n = 51) and for other/unknown reasons (n = 6). Our final analysis was conducted in a subset of 2606 participants for whom both microRNA expression data and cardiovascular measurements were available.

R5.4: *All clinical assessments, such as blood pressure measurement and the formula for calculating mean arterial pressure (MAP), should be referenced for transparency.*

Answer:
We have added the following references for MAP and Omron 705 IT:

- Mean arterial pressure:
    - Hall JE. Guyton and hall textbook of medical physiology. 13th edition. London, England: W B Saunders; 2015 [#24]
- Omron 705 IT:
    - Coleman, A., et al. Validation of the Omron 705IT (HEM-759-E) oscillometric blood pressure monitoring device according to the British Hypertension Society protocol. Blood Press Monit 11, 27-32 (2006) [#22]
    - El Assaad, M.A., Topouchian, J.A. & Asmar, R.G. Evaluation of two devices for self-measurement of blood pressure according to the international protocol: the Omron M5-I and the Omron 705IT. Blood Press Monit 8, 127-133 (2003) [#23]

Additionally, we have revised the mean arterial pressure (MAP) section to incorporate the formula for calculating MAP, replacing the use of words. This method is the most common one to calculate MAP:

Line 180: Mean arterial pressure (MAP) was calculated as $(SBP + 2 \times DBP)/3$.

R5.5: *While the "Omron 705 IT" device was chosen for specific reasons, it's essential to note its limitations compared to conventional mercury sphygmomanometers, which are considered the gold standard. Factors like cuff size, position, arm position, side of measurement, and personnel performing the measurement need to be addressed.*

Answer: Omron 705 IT machine was used instead of manual mercury sphygmomanometers because automated blood pressure devices are user independent and provide accurate measurements. We also included references comparing our device to mercury sphygmomanometers, showing that it provides comparable results and is approved for use in professional settings.

Additionally, we have included the following details in the blood pressure section:

Line 170: Systolic blood pressure (SBP) and diastolic blood pressure (DBP) were measured in a sitting position, using an oscillometric blood pressure device (Omron 705 IT) [22, 23]. The measurements were performed thrice, separated by ten minutes, by experienced study technicians, while participants were sitting in a resting chair in a quiet environment. Cuff size was determined by measuring participants' arm circumference in

the middle of the upper arm between the acromion and olecranon on the right arm of the participant sitting in the measuring position. Measurements were preferably performed in the right arm. In cases where the measurements were not possible on the right arm, the left arm was used. The measured arm was always placed in a resting position at heart level, with the palms facing upward, the shoulders in a horizontal position, and both legs resting on the ground. The mean of the second and third measurement was used in the analyses. Mean arterial pressure (MAP) was calculated as (SBP + 2 × DBP)/3.

R5.6: *Justifying why central venous pressure was considered "zero" in the study is necessary.*

Answer: We set central venous pressure (CVP) at 6 mmHg as recommended by the producer. A normal range of CVP may vary from 5-15 mmHg and might be affected by different factors such as underlying cardiac, hepatic and pulmonary diseases, as well as volume load and breathing pressures (*Shah P, Louis MA. Physiology, Central Venous Pressure. 2023 Jul 10. In: StatPearls [Internet]. Treasure Island (FL): StatPearls Publishing; 2024 Jan–. PMID: 30137777*, *Oh, C., Noh, C., Hong, B. et al. Is measurement of central venous pressure required to estimate systemic vascular resistance? A retrospective cohort study. BMC Anesthesiol 21, 310 (2021))*.
As central venous pressure is measured with a very invasive technique (right heart catheterization), most of the data available to date is based on patient cohorts and the reference range for the general population may be lacking. However, a study has suggested a median value of 7 mmHg for central venous pressure in spontaneously breathing patients in the supine position (*Prekker, M. E., Scott, N. L., Hart, D., Sprenkle, M. D., & Leatherman, J. W. (2013). Point-of-care ultrasound to estimate central venous pressure: a comparison of three techniques. Critical care medicine, 41(3), 833–841*).
Moreover, a recent study has shown that setting CVP at certain values does not impact systemic vascular resistance-related outcomes in patients (*Oh, C., Noh, C., Hong, B. et al. Is measurement of central venous pressure required to estimate systemic vascular resistance? A retrospective cohort study. BMC Anesthesiol 21, 310 (2021)*).

We now included this in the methods as follows:

Line 189: We set central venous pressure (CVP) at 6 mmHg as recommended by the producer. Whereas normal CVP reportedly can vary from 5-15 mmHg [26], a recent study has shown that setting CVP at certain values does not impact systemic vascular resistance-related outcomes [26].

R5.7: *The definition of stroke volume needs to be clearly stated.*

Answer: Based on your comment, we have slightly adapted the definition of stroke volume in our manuscript:

Line 193: Stroke volume (mL) was defined as the volume of blood pumped from the left ventricle to systemic circulation in each heartbeat and computed using beat-to-beat for approximately 8 minutes with an impedance cardiography device (CardioScreen 2000, Medis, Germany) [27].

R5.8: *There's a lack of methodology provided for how impedance cardiography was performed.*

Answer: To expand this session and provide more details, we have added the following sentences in the hemodynamic parameters section with the corresponding reference:

Line 183: Impedance cardiography was performed with the CardioScreen 2000 device (Medis, Germany), by experienced study technicians, in a temperature-controlled room. Before the examinations, the participants were placed in a supine position and allowed to rest for five minutes. Electrodes as well as arm, ankle and thigh cuffs were placed as per the device manufacturer's recommendations [25]. All hemodynamic measurements were calculated by in-developed software, based on simultaneously registered electrocardiography signals and blood pressures with 2-minute intervals.

R5.9: *The rationale behind not using echocardiography should be explained.*

Answer: Impedance cardiography device was preferred to echocardiography because the device is user independent and it provides data of 8 minutes measurements. On the other hand, echocardiography is heavily user-dependent and can only provide measurements from three consequent heartbeats (*Lorne E, Mahjoub Y, Diouf M, Sleghem J, Buchalet C, Guinot PG, Petiot S, Kessavane A, Dehedin B, Dupont H. Accuracy of impedance cardiography for evaluating trends in cardiac output: a comparison with oesophageal Doppler. Br J Anaesth. 2014 Oct;113(4):596-602*).
Moreover, echocardiography is not the gold standard to measure most of the hemodynamic measurement, particularly stroke volume, which is typically assessed by transpulmonary thermodilution (TPTD) (*Pugsley, J., & Lerner, A. B. (2010). Cardiac output monitoring: is there a gold standard and how do the newer technologies compare? Seminars in cardiothoracic and vascular anesthesia, 14(4), 274–282*).
Finally, hemodynamic measurements assessed with impedance cardiography are associated with mortality in the general population (*Medina-Lezama J et al. Hemodynamic Patterns Identified by Impedance Cardiography Predict Mortality in the General Population: The PREVENCION Study. J Am Heart Assoc. 2018 Sep 18;7(18):e009259*).

R5.10: *The accuracy and validity of the methods used in the study are not discussed, which is crucial for ensuring the reliability of the results.*

Answer: Based on your comments and suggestions, we have revised the sections regarding blood pressure and hemodynamic measurements. This revision includes additional detailed information about the measurements and their references. We hope that this helps improve the assessment of the accuracy and validity of the methods.

R5.11: *Indexing hemodynamic parameters to body surface area is a common practice, but it's important to acknowledge its limitations. The assumption of a linear relationship between body size and hemodynamic parameters may not always hold true, especially in populations with diverse body compositions or morphologies.*

Answer: We agree that indexing hemodynamic parameters have limitations. To partially address this, we conducted a sensitivity analysis exploring the relationship between microRNAs and non-indexed cardiovascular measurements, additionally adjusting for

BMI. Our findings revealed similar but weaker associations compared to those observed using indexed-cardiovascular measurements (Line 454). The sensitivity analysis confirmed the importance of the candidate microRNAs associated with indexed-cardiovascular measurements in vascular health.

R5.12: *The choice of the formula used to calculate body surface area should be justified, considering its applicability across different populations.*

Answer: The body surface area is calculated with DuBois formula, which is the most common method used worldwide (*Du Bois D, Du Bois EF. A formula to estimate the approximate surface area if height and weight be known. 1916. Nutrition. 1989;5(5):303-11; discussion 12-3*). We have also cited the Du Bois paper in the manuscript.

R5.13: *There's a need to be cautious when interpreting indexed hemodynamic parameters and to consider the impact of body composition and population-specific factors.*

Answer: Thank you for the comment. Body composition and population-specific factors may indeed influence the hemodynamics parameters. Since components of cardiovascular system, like heart chambers and large arteries such as the aorta, are correlated to body size, using hemodynamics indexed to body surface area accounts to some extent for the confounding effects of factors such as sex, obesity and body size. Moreover, in the sensitivity analysis we compared the effect estimates derived from associations between microRNAs and indexed-cardiovascular measurements to those obtained using non-indexed parameters. Given that the results were comparable, we consider that body composition does not substantially influence the associations we found.

We agree that we should address this in our manuscript as a limitation:

Line 691: Fourth, we evaluated indexed hemodynamic parameters, which is a standard approach in daily clinical practice and cardiovascular research. Components of the cardiovascular system, like the heart chambers and large arteries, are correlated to body size. Using hemodynamic parameters indexed to body surface area accounts to some extent for confounding due to factors such as sex, obesity and body size. However, we did not account for body composition and population-specific factors. While it is possible that these factors also influence the specific estimates of the parameters we investigated, we consider it unlikely that this would completely alter our findings. Moreover, the sensitivity analysis performed using non-indexed parameters revealed effect estimates comparable to those obtained using indexed-cardiovascular measurements, suggesting that body composition does not substantially influence the associations we identified.

R5.14: *Rationalizations behind the chosen parameters and their insights into vascular function and disease pathophysiology are missing, which is essential for understanding the significance of the study findings.*

Answer: We aimed to investigate the link between microRNAs and pulsatile and steady vascular parameters, which have been previously shown to be associated with sub- and

clinical alterations in population-based studies. These parameters are linked to risk of mortality and cardiovascular diseases.
